# A chikungunya virus-like particle vaccine reduces chikungunya disease in cynomolgus macaques and protection is mediated by antibody transferred from vaccinated humans

**DOI:** 10.1038/s41541-026-01413-z

**Published:** 2026-03-18

**Authors:** Lark L. Coffey, Katherine J. Olstad, J. Rachel Reader, Amir Ardeshir, Christopher M. Weiss, Jennifer K. Watanabe, Jodie L. Usachenko, JoAnn Yee, Anil Singapuri, Zhong Min Ma, Alexis Mackiewicz, Rebecca Sammak, Jackson Stuart, Ramya Immareddy, Ravi Anantha, Kelly L. Warfield, Darly Manayani, Jeff Alexander, Jonathan Smith, Lo Vang, Christopher M. Cirimotich, Cassandra O’Connor, Ben Guenther, Nhuxuan Ho, Christopher S. Morello, Jason Mendy, Jason S. Richardson, Koen K. A. Van Rompay

**Affiliations:** 1 Department of Pathology, Microbiology, and Immunology, Weill School of Veterinary Medicine, University of California, Davis, Davis, CA USA; 2 California National Primate Research Center, University of California, Davis, Davis, CA USA; 3 Emergent Biosolutions, Gaithersburg, MD USA; 4 Sabin Vaccine Institute, Washington, DC USA; 5 Novartis, San Diego, CA USA; 6 VLP Therapeutics, Gaithersburg, MD USA; 7 Bavarian Nordic, San Diego, CA USA; 8 Battelle Biomedical Research Center, West Jefferson, OH USA; 9 Bavarian Nordic, Toronto, ON Canada; 10 Present Address: Tulane National Biomedical Research Center, Covington, LA USA; 11 Present Address: MRIGlobal, Kansas City, MO USA; 12 Present Address: Forge Biologics Inc, Grove City, OH USA

**Keywords:** Diseases, Immunology, Microbiology

## Abstract

Chikungunya virus (CHIKV) causes periodic outbreaks and is endemic in more than 110 countries. VIMKUNYA, a CHIKV virus-like particle (CHIKV VLP) vaccine, was recently approved by regulators in the United States, European Union, and United Kingdom. Efficacy of VIMKUNYA in endemic settings is difficult to evaluate due to outbreak unpredictability. We used cynomolgus macaques, which model human CHIKV viremia and disease, to assess CHIKV VLP vaccine efficacy. Doses as low as 1.25 μg of CHIKV VLP with aluminum hydroxide adjuvant and passively transferred IgG from vaccinated humans significantly reduced viremia, disease, and joint pathology. Animals that received IgG doses resulting in mean reciprocal 80% neutralization titers of 35, well below the predicted protective threshold of ≥100, exhibited improved clinical outcomes compared with CHIKV-infected control animals, suggesting clinical benefits may occur at lower antibody levels. These findings demonstrate immunogenicity and protective efficacy of CHIKV VLP and relevance of neutralizing antibodies in protection, reinforcing its use in humans to protect against chikungunya disease.

## Introduction

Chikungunya virus (CHIKV, family *Togaviridae*, genus *Alphavirus*, species *chikungunya*) is an emerging mosquito-borne pathogen that causes a febrile illness characterized by headache, myalgia, and arthralgia, the latter of which can be chronic and debilitating in up to 60% of patients^1,2^. CHIKV comprises three major genetic lineages: West African, East/Central/South African (ECSA, which includes the Indian Ocean Lineage [IOL]), and Asian^3^, but only a single serotype. Following infection, CHIKV causes an acute viremia that disseminates to muscle, joint, and lymphoid tissues^4^. CHIKV-specific neutralizing antibodies play a critical role against chikungunya (CHIK) disease by mediating early control of infection and providing protection against disease at re-infection^4–9^. CHIKV is currently endemic in more than 110 countries spanning Africa, Asia, the Indian Ocean and South Pacific islands, Europe, and the Americas, with over 20 million cases since 2005, of which nearly 4 million occurred in the Americas^10,11^. From mid‑2022 through mid‑2025, there have been ~1.2 million CHIK disease cases reported globally, particularly due to recent outbreaks in Brazil^12^, Reunion Island^13,14^ and China^15^. In October 2025, a locally acquired case of CHIK disease was reported in New York^16^, highlighting the threat of CHIKV to the United States. The development of safe and effective CHIKV vaccines is therefore a pressing global health need.

In 2023 and 2025, two CHIKV vaccines received regulatory approval for prevention of CHIK disease: a live-attenuated vaccine (VLA1553, marketed as IXCHIQ®) that was approved in individuals 18 and older, and a virus-like particle (VLP) vaccine (PXVX0317, marketed as VIMKUNYA™), that was approved in individuals 12 and older. These vaccines were approved by the U.S. Food and Drug Administration (US FDA) and, in some cases, by Canadian, European, and Brazilian regulators, with additional authorizations pending^17–21^. IXCHIQ approval was suspended for all ages by the US FDA in August 2025 due to serious adverse events in post-licensure evaluations^22^; in response, the manufacturer, Valneva, issued a public statement affirming its commitment to continuing to provide vaccine access and ensuring vaccine safety, concluding that the vaccine’s benefit-risk profile did not outweigh its risks and that serious adverse events are under investigation^23^. Both vaccines were extensively tested in pre-clinical and clinical trials prior to approval (reviewed in ref. ^24^). The unadjuvanted CHIKV VLP vaccine was initially developed by the US National Institutes of Health Vaccine Research Center and was subsequently manufactured and reformulated with aluminum hydroxide adjuvant (PXVX0317). PXVX0317 consists of the recombinant CHIKV structural proteins—capsid, envelope (E) 2, and E1—derived from the Senegalese CHIKV strain 37997 (West African genotype). When expressed in vitro, these proteins self-assemble into non-replicating VLPs that closely resemble wild-type CHIKV in structure but lack a viral genome, making them replication-incompetent^24^. The CHIKV VLPs are therefore unable to infect or cause disease. The proposed mechanism of action for PXVX0317 is the induction of neutralizing antibodies against the CHIKV capsid, E2, and E1 proteins to limit CHIKV infection^25^. Preclinical studies in mice and rhesus macaques demonstrated that the CHIKV VLP was immunogenic and efficacious^24^. Mice that received IgG purified from vaccinated macaques by passive transfer did not develop detectable viremia and were protected from lethal disease, underscoring the protective role of vaccine-induced IgG against CHIK disease.

Safety and immunogenicity of the CHIKV VLP in five clinical trials (ClinicalTrials.gov NCT04189358, NCT02562482, NCT03483961, NCT05072080, NCT05349617) at sites across the U.S. and in CHIKV-endemic regions of Latin America demonstrated safety, tolerability, and immunogenicity of CHIKV VLP, and informed both dose selection and adjuvant formulation^26–30^; reviewed in ref. ^23^). However, due to the unpredictability of CHIKV outbreaks, real-world efficacy data in areas where CHIKV is endemic are lacking^31^.

Building on the initial promising CHIKV VLP immunogenicity and efficacy data^25^, we sought to further evaluate this vaccine by quantifying protection from clinical disease, testing adjuvanted formulations, assessing protection mediated by vaccine-induced human IgG, and confirming cross-genotype efficacy not addressed in the earlier work. To evaluate efficacy of the CHIKV VLP against CHIK disease, we employed a non-human primate (NHP) model, which is susceptible to CHIKV and exhibits viremia, musculoskeletal tropism, and disease signs similar to humans^32^ and has been used previously to study CHIKV pathogenesis^33^ and to evaluate vaccine efficacy^9^^,^^34–37^. In particular, cynomolgus macaques challenged with 10^7^ plaque forming units (PFU) of the CHIKV IOL strain LR2006-OPY1 develop measurable viremia and arthritis-related manifestations^38^ that mimics severe arthritic CHIK disease in humans. Prior NHP studies showed a dose-dependent relationship between inoculation levels and viremia and clinical outcomes: intermediate doses (10^2^–10^6^ PFU) caused viremia, fever, and rash, while higher doses (≥10^7^ PFU) also resulted in joint effusion, and at 10^8^ PFU, meningoencephalitis and death^38^.

Guided by these findings, our Study A aimed to identify a CHIKV challenge dose that consistently induces high viremia, clinical signs of disease, and histopathologic changes in muscles and joints resembling typical human CHIK disease without high risk of lethality. We tested 3 challenge doses (10^6^, 10^7^, and 10^8^ PFU) of CHIKV strain LR2006-OPY1 in cynomolgus macaques. Study B evaluated the immunogenicity and protective efficacy of both unadjuvanted and aluminum hydroxide adjuvanted formulations of CHIKV VLP. In Study C, we assessed the protective efficacy of CHIKV-specific IgG purified from human clinical trial participants vaccinated with CHIKV VLP. Efficacy in Studies B and C was assessed by challenging both vaccinated and control animals (those receiving aluminum hydroxide adjuvant alone or control IgG from unvaccinated humans) with a single challenge dose of CHIKV strain LR2006-OPY1, as determined from Study A. The CHIKV challenge strain, LR2006-OPY1, belongs to the ECSA genotype and was selected to assess vaccine efficacy against a heterologous genotype, as the CHIKV VLP is derived from a West African genotype strain, despite all CHIKV genotypes comprising a single serotype^39^. After challenge, we assessed viremia, CHIKV RNA levels in tissues, clinical signs of disease, and histopathologic signs of inflammation. By testing various doses of vaccine and passively transferred IgG, we were able to investigate vaccine efficacy and IgG as a pharmacodynamic marker of protection in a model of severe CHIK disease.

## Methods

### Animal acquisition and housing, screening and quarantine, and animal care accreditation

All animals used in the study were adult (4.7–9.2 years of age) male and female cynomolgus macaques (*Macaca fascicularis*) with weights on study day 0 ranging from 2.8 to 8.3 kg obtained from Valley Biosystems (Sacramento, CA, USA). Before study enrollment at the California National Primate Research Center (CNPRC), all animals were screened for CHIKV-VLP specific IgG antibodies using an enzyme linked immunosorbent assay (ELISA); animals with background CHIKV-VLP specific IgG ELISA signals were excluded. The CNPRC is accredited by the Association for Assessment and Accreditation of Laboratory Animal Care International (AAALAC), and animal care followed the 2011 *Guide for the Care and Use of Laboratory Animals* from the Institute for Laboratory Animal Research. Study protocol (#20249) and amendments were approved by the UC Davis Institutional Animal Care and Use Committee (IACUC). Upon arrival at CNPRC, each animal received a CNPRC identification number, which was tattooed. They were housed indoors in stainless steel cages (Lab Products Inc., Seaford, DE), with cage sizes adjusted according to national standards. Housing conditions included a 12-hour (h) light/dark cycle, temperatures between 64 and 84 °F, and relative humidity between 30 and 70%. Animals previously housed together were kept paired in housing at CNPRC. They had *ad libitum* access to water and were fed a commercial high-protein diet (Ralston Purina Co., St. Louis, MO) twice daily, supplemented with fresh produce. Animals were fasted overnight prior to sedation for procedures. Animal identity was verified prior to all procedures. Animals underwent a 90-day quarantine period that included physical exams, sample collection, tuberculosis testing, and other health assessments. Initially housed in an animal biosafety level 2 (ABSL-2) facility, animals were transferred into an animal biosafety level 3 (ABSL-3) prior to CHIKV challenge, where they remained until the study endpoint at 10 days post-challenge. The placebo animal remained in ABSL-2 for the entire study.

### Study groups

This project included three studies and a total of 58 adult male and female cynomolgus macaques evenly distributed by sex (Table 1). In Study A, we evaluated clinical signs of disease and viremia kinetics following three different challenge doses of CHIKV strain LR2006-OPY1 with the goal of identifying a suitable dose for use in subsequent studies. In Study B, we assessed the immunogenicity and protective efficacy of CHIKV VLP vaccine formulations, both with and without aluminum hydroxide adjuvant. In Study C, we evaluated the protective efficacy of CHIKV-specific IgG purified from human participants in CHIKV VLP clinical trials.

**Table 1:** Cynomolgus macaques enrolled in the study

Study	Animal identifier	Sex	Age at start of study in years	Treatment
A	A01	F	8.0	10^6^ PFU CHIKV
	A02	F	8.3	10^6^ PFU CHIKV
	A03	M	8.0	10^6^ PFU CHIKV
	A04	F	5.6	10^7^ PFU CHIKV
	A05	F	5.2	10^7^ PFU CHIKV
	A06	M	7.1	10^7^ PFU CHIKV
	A07	F	7.3	10^8^ PFU CHIKV
	A08	F	7.2	10^8^ PFU CHIKV
	A09	M	5.9	10^8^ PFU CHIKV
	A10	M	7.1	placebo
B	B01	F	5.6	1.25 ug VLP + alum
	B02	M	6.1	1.25 ug VLP + alum
	B03	M	7.4	1.25 ug VLP + alum
	B04	F	8.2	1.25 ug VLP + alum
	B05	M	7.8	1.25 ug VLP + alum
	B06	F	9.2	6 ug VLP + alum
	B07	F	9.1	6 ug VLP + alum
	B08	M	4.9	6 ug VLP + alum
	B09	M	5.0	6 ug VLP + alum
	B10	M	8.8	6 ug VLP + alum
	B11	M	8.2	20 ug VLP + alum
	B12	M	8.1	20 ug VLP + alum
	B13	F	8.4	20 ug VLP + alum
	B14	F	8.2	20 ug VLP + alum
	B15	M	7.3	20 ug VLP + alum
	B16	F	8.4	20 ug VLP
	B17	M	4.7	20 ug VLP
	B18	M	7.6	20 ug VLP
	B19	M	8.3	20 ug VLP
	B20	F	8.5	20 ug VLP
	B21	M	6.9	alum
	B22	F	8.6	alum
	B23	M	6.3	alum
	B24	F	9.1	alum
C	C01	F	6.7	5 mg/kg VLP vaccinee IgG
	C02	M	5.8	5 mg/kg VLP vaccinee IgG
	C03	F	6.6	5 mg/kg VLP vaccinee IgG
	C04	M	5.8	5 mg/kg VLP vaccinee IgG
	C05	F	5.5	5 mg/kg VLP vaccinee IgG
	C06	M	5.7	5 mg/kg VLP vaccinee IgG
	C07	F	7.1	15 mg/kg VLP vaccinee IgG
	C08	M	5.8	15 mg/kg VLP vaccinee IgG
	C09	F	6.5	15 mg/kg VLP vaccinee IgG
	C10	M	6.3	15 mg/kg VLP vaccinee IgG
	C11	F	6.5	15 mg/kg VLP vaccinee IgG
	C12	M	5.8	15 mg/kg VLP vaccinee IgG
	C13	F	7.1	100 mg/kg VLP vaccinee IgG
	C14	M	5.6	100 mg/kg VLP vaccinee IgG
	C15	F	6.7	100 mg/kg VLP vaccinee IgG
	C16	M	5.7	100 mg/kg VLP vaccinee IgG
	C17	F	7.3	100 mg/kg VLP vaccinee IgG
	C18	M	5.8	100 mg/kg VLP vaccinee IgG
	C19	F	5.2	100 mg/kg VLP naive IgG
	C20	M	5.8	100 mg/kg VLP naive IgG
	C21	F	6.4	100 mg/kg VLP naive IgG
	C22	M	5.8	100 mg/kg VLP naive IgG
	C23	F	7.3	100 mg/kg VLP naive IgG
	C24	M	5.7	100 mg/kg VLP naive IgG

#### Study A

This study included 10 animals. Three groups of three animals each were inoculated intravenously (IV) with different doses (10^6^, 10^7^, or 10^8^ PFU) of CHIKV LR2006-OPY1. These doses exceed CHIKV levels that *Aedes aegypti* vectors deposit into collection tubes^33^, a laboratory proxy for transmission, and mosquito probing can occur both in venules and in the epidermis^40^. However, these levels and the IV route were selected to ensure consistent infection and to elicit signs of arthritic disease that are not reliably observed with lower challenge doses or subcutaneous routes of inoculation. One animal received a placebo consisting of growth medium (Dulbecco’s Modified Eagle’s Medium [DMEM]) with fetal bovine serum (FBS) and antibiotics. To mirror some of the groups in Study B, all animals received aluminum hydroxide adjuvant approximately 8 weeks prior to CHIKV challenge.

#### Study B

This study included 24 animals divided into five groups. Four groups of five animals each received different doses (1.25, 6, or 20 μg) of the CHIKV VLP with 300 μg aluminum hydroxide adjuvant administered intramuscularly (IM) twice, or 20 μg unadjuvanted CHIKV VLP, on study days 0 and 28. A fifth group of four animals received 300 μg aluminum hydroxide adjuvant only. On study day 56, all animals were subsequently challenged IV with 10^7^ PFU CHIKV LR2006-OPY1.

#### Study C

This study included 24 animals. Three groups of six animals each were IV administered different doses (5, 15, or 100 mg/kg body weight) of IgG purified from humans vaccinated with the CHIKV VLP. A fourth group of six animals received 100 mg/kg IgG from unvaccinated (VLP naïve) humans. One day later, all animals were challenged IV with 10^7^ PFU CHIKV LR2006-OPY1.

### Administration of agents, sedation, sample collection, and clinical monitoring

Animals were sedated with ketamine hydrochloride (Parke Davis, Detroit, MI) at 10 mg/kg injected IM after overnight fasting for all procedures including vaccination, IgG administration, CHIKV challenge, and blood collection. Blood samples were collected via venipuncture. Prior to CHIKV challenge, animals were monitored at least once daily for clinical signs of illness such as behavioral changes, reduced appetite, abnormal stool, altered mobility, or other health concerns. Any signs of illness were recorded at the time of observation and evaluated by a veterinarian. Following CHIKV challenge, animals were monitored twice daily, or more frequently if they showed reduced mobility or reluctance to move, for 10 days. During the first week post-challenge, animals were sedated daily for blood collection and underwent physical examination, which included measurements of body weight and rectal temperature, assessment of rash, and evaluation of joints (stifle, ankle, and wrist) for signs of joint effusion. Based on clinical signs, and following IACUC protocol guidelines for alleviating animal discomfort, veterinary staff made independent decisions to treat with analgesics. Analgesics that were used for supportive care included ketoprofen (5 mg/kg once daily administered IM) and long-acting buprenorphine (Simbadol, 0.72 mg/kg, injected subcutaneously every 3 days).

### Preparation and administration of adjuvant, CHIKV VLP, and human IgG

In Study A, aluminum hydroxide gel (Alhydrogel® 2%, ‘alum’, Brenntag, Lot No.5427) was administered IM at 300 µg per animal in single dose volume of 0.8 ml, injected into the right quadriceps on study day 0. In Study B, the CHIKV VLP vaccine was prepared using three components: CHIKV VLP (Lot Number 1-FIN-2949), aluminum hydroxide (same lot as above), and a diluent matching the excipient composition of the VLP drug product (Lot No. 1-FIN-2985). Each 0.8 ml dose was administered via IM injection on study day 0 in the right quadriceps muscle and on study day 28 in the left quadriceps. In Study C, purified human IgG was used. The purified human IgG was obtained from participants enrolled in two previous Phase II clinical trials (NCT02562482 and NCT03483961). Both clinical trials were conducted in compliance with approved protocols, Good Clinical Practice guidelines, and all applicable regulatory requirements. Each trial received Institutional Review Board approval, and written informed consent was obtained from all participants prior to sample collection. The purified IgG included a sterile buffered aqueous IgG solution (Lot No.PD_740_CDM_19_001_001) containing 0.03% polysorbate 80 (w/w) and 10% maltose (w/w), pH 5.5 ± 0.15. IgG from CHIKV VLP vaccinees was supplied at 80 mg/ml, while control IgG from VLP naïve humans (Lot No. PD_740_CDM_19_001_003) was at 105 mg/ml. The IgG from CHIKV VLP vaccinees was derived from an IgG preparation made from the plasma of 8 study participants obtained by plasmapheresis 28 days after the receipt of the second of two 20 µg CHIKV VLP with aluminum hydroxide doses given 28 days apart. IgG was purified using a scaled down, validated human hyperimmune gamma globulin purification platform at Emergent BioSolutions Canada Inc. The formulated CHIKV VLP IgG was determined to contain >99.5% IgG monomer/dimer by size-exclusion chromatography and a serum neutralizing antibody geometric mean titer (GMT) of 15,026 (range 11,790 to 19,151). The control IgG was derived from a pool of sera collected prior to vaccination from pre-immune clinical trial participants who were baseline CHIKV seronegative. The diluent (Lot No. B170-00943) matched the excipient composition of the IgG solution. All reagents were provided by Emergent BioSolutions Canada Inc. and stored at 2–8 °C prior to use. For administration, IgG was injected IV in the right saphenous vein on study day 0, ~24 h prior to CHIKV challenge. Control IgG and higher-dose IgG (100 mg/kg and 15 mg/kg) were administered undiluted. For the lowest dose (5 mg/kg), the IgG was diluted 3-fold before adjusting the volume to match the weight of each animal.

### Preparation and administration of the CHIKV stock

A challenge stock of CHIKV strain LR2006-OPY1 (GenBank accession number DQ443544.2), originally isolated from a human in 2006 in Réunion Island, France, was used. The virus had undergone four passages on African green monkey (Vero; ATCC CCL-81) cells before being lyophilized. Lyophilized virus was obtained from Dr. Scott Weaver at the World Reference Center for Arboviruses (Galveston, TX, USA), then propagated once more in Vero cells to reach high titer and was verified by sequencing. The Vero cells were grown in Dulbecco’s modified Eagle’s medium (DMEM) (Gibco 11965-092 [Lot 1967577]) supplemented with 10% heat inactivated FBS (56 °C for 30 min) (GenClone 25-514H [Lot P073168]) and 1X penicillin and streptomycin from a stock concentration of 10,000 IU (Gibco 15140-122 [Lot 1953101]). This stock was diluted to prepare target challenge doses of 10^6^, 10^7^, and 10^8^ PFU and stored at −80 °C. Vials of each dilution were then thawed and titrated to determine the actual titer. The challenge stocks for each dose were transferred to the CNPRC ABSL-3 facility for storage at −80 °C prior to challenge studies. For each animal, a separate vial of the appropriate dilution was thawed rapidly in a 37 °C water bath just prior to inoculation and kept on wet ice until administration. CHIKV was administered IV in the left saphenous vein. The remaining inoculum was re-frozen at −80 °C for subsequent back-titration.

### Quantitation of CHIKV RNA in plasma and tissues

For Study A, CHIKV RNA levels in plasma and tissue samples were measured at the University of California, Davis using a quantitative reverse transcription PCR (RT-qPCR) assay based on the method described by Lanciotti^41^ using CHIKV 6856 forward primer: TCACTCCCTGTTGGACTTGATAGA, CHIKV 6981 reverse primer: TTGACGAACAGAGTTAGGAACATACC, and CHIKV 6919-FAM probe: 5’FAM AGGTACGCGCTTCAAGTTCGGCG BHQ1. Whole blood was collected in EDTA-anticoagulated blood tubes and processed immediately. The blood was centrifuged at 800 × *g* for 10 minutes (min) to separate plasma, which was spun again at 800 × *g* for another 10 min to remove residual cells. Plasma aliquots were immediately frozen at −80 °C. Tissues were placed in 2.0 ml round bottom tubes with a 5 mm steel bead and 250 µl DMEM. Tubes were weighed before and after tissue addition to determine tissue weight and then frozen at −80 °C until later analysis. Tissues were homogenized for 2 min at 30 shakes per second using a Mixer Mill (Qiagen, Germantown, MD). The homogenate was centrifuged at 14,000 × *g* for 2 min to clarify the supernatant. If tissue was not fully liquified, samples were re-homogenized for an additional 2 min; if needed, 250 µl of DMEM was added and the sample was weighed and homogenized again. RNA was extracted from 140 µl of plasma or homogenized tissue supernatant using a viral RNA isolation kit and machine (MagMAX, Waltham, MA). For each sample, 200 µl was mixed with 10 µl RNA binding beads, 10 µl of lysis binding enhancer, 120 µl of 100% isopropanol (Geel, Antwerp, Belgium) and 120 µl of lysis binding solution. Samples were processed in a 96-well deep plate format. RNA was eluted in 60 µl of DEPC-treated water (ThermoFisher, Waltham, MA) and stored at −80 °C until analysis. All samples were tested in triplicate, and results were averaged. CHIKV RNA levels are reported as mean log_10_ RNA copies per gram of tissue or per milliliter of plasma. For Studies B and C, CHIKV RNA levels in plasma were measured at Battelle Biomedical Research Center (West Jefferson, OH). RNA from each plasma sample (ca. 200 µL) was extracted together with a spiked internal control (IC) consisting of *Escherichia coli* bacteriophage MS2 (ATCC 15597-B1) diluted in BaseMatrix (SeraCare 1805-0075). Extractions were performed using QIAamp 96 Virus Kits (#57731) and a QIAcube HT extraction system (Qiagen, Germantown, MD). Each extraction batch included a negative control and high and low positive controls containing the CHIKV gene target at different concentrations generated by spiking CHIKV LR2006-OPY1 stock into naïve NHP plasma. RNA samples were eluted in buffer and stored at ≤−60 °C until RT-qPCR analysis. RT-qPCR was performed using the QuantStudio 6 Flex Real-time PCR system (ThermoFisher, Waltham, MA) and SuperScript III Platinum One-Step Quantitative RT-PCR system (ThermoFisher 11732-088) as the Master Mix. RT-qPCR analysis of each sample comprised of 1) CHIKV RNA copy quantification using a qualified CHIKV reference standard (RS) dilution series and 2) an MS2-specific RT-qPCR qualitative assay for detection of IC to confirm RNA extraction efficiency, RNA quality, and lack of RT-qPCR inhibition. The CHIKV RS consisted of an 8-point dilution of a 173-nucleotide synthetic RNA (Bio-Synthesis, Lewisville, TX) derived from strain LR2006-OPY1 genome positions 6832–7004 (5’-GCUGUUAGAG…AUCACCAUCG-3’). For each sample, the reportable CHIKV RNA copies/ml value was calculated from the mean RNA copy results from duplicate wells, reaction input volume, RNA eluate volume, and actual volume of plasma extracted. CHIKV RNA copy data were reportable if the MS2 reaction for the test sample resulted in a C_T_ value ≤ 3 C_T_s lower than that of the CHIKV RNA negative/MS2 spiked extraction control for its respective extraction batch. Prior to study use, the CHIKV and MS2 assays together with RNA extraction procedure were validated for use on NHP plasma samples using qualified assay components. The validated CHIKV assay lower limit of detection (LOD) and lower limit of quantification (LLOQ) were 292 and 1060 CHIKV RNA copies/ml of plasma, respectively.

### Quantification of infectious CHIKV in plasma and inoculum back-titration

Infectious CHIKV levels in plasma and inoculum stocks were determined using Vero cell plaque assay. Vero cells were seeded into tissue culture plates and grown to 100% confluence at 37 °C with 5% carbon dioxide. Plasma samples were serially 10-fold diluted in DMEM and tested in technical duplicates. Each dilution was added to the cell monolayers and incubated for 1 hour (h), with rocking every 10 min. After the 1 h incubation, 0.4% agarose (Genesee Scientific, El Cajon, CA) in DMEM maintained at 42 °C was added to each well. Plates were incubated for 3 days at 37 °C with 5% carbon dioxide to allow plaque formation. Cells were then fixed with 2% formalin for 1 h, after which the agarose overlay was removed. Plates were stained with 0.05% w/v crystal violet (Millipore-Sigma, Burlington, MA) for 5 min, rinsed with deionized water, and dried before plaques were counted. Viral titers were calculated as PFU/ml based on visual counts of plates placed on a lighted box. Titers were determined based on plaque counts from one or more wells in the dilution series. If plaques were counted from multiple wells, the average was used. When plaques were detected in repeated titrations, the average of the results with the lower standard deviation was used. The maximum LOD is represented as a dotted line on graphs; values below this line reflect samples tested with a lower LOD. LOD varied depending on the sample volume available and the size of the titration plate.

### Complete blood counts (CBC)

CBC were conducted on EDTA-anticoagulated blood samples. Total cell counts were measured using the VET ABC system (SCIL Animal Care, Gurnee, IL), following the manufacturers’ instructions. Differential cell counts were performed by the CNPRC Clinical Laboratories using Giemsa or Wright-Giemsa staining.

### Blood chemistry

Serum biochemistry analysis was conducted using Piccolo BioChemistry Plus panels on the Piccolo Xpress chemistry analyzer (Union City, CA), according to the manufacturer’s protocol. This panel measures the following parameters: alanine aminotransferase (ALT), albumin, alkaline phosphatase (ALP), amylase, aspartate aminotransferase (AST), C-reactive protein, calcium, creatinine, gamma glutamyltransferase (GGT), glucose, total protein, blood urea nitrogen (BUN), and uric acid.

### Neutralizing antibody assays

Neutralizing antibody titers (NT) in serum samples were assessed using a CHIKV-Luciferase (Luc) neutralization test at 80% reduction (NT_80_) assay with CHIKV strain 181/25 (from the heterologous [Asian genotype] to the CHIKV VLP, GenBank accession number AF15561) expressing a firefly luciferase enzyme. Serial dilutions of heat-inactivated macaque serum were incubated with a known amount of CHIKV-Luc for 90 min. These virus-serum mixtures were then added to Vero cell monolayers in 96-well tissue culture plates and incubated for 20 h. Controls included sera with pre-established negative, low, medium, and high NT_80_ values. Luciferase expression, which reflects viral infection levels, was measured using a microplate luminometer. The NT_80_ were defined as the reciprocal serum dilution that reduced luciferase activity by 80% compared to virus-only controls after background subtraction and linear interpolation. Prior to use, the assay was validated at Emergent BioSolutions Inc. for use with NHP serum samples using previously qualified NHP serum controls, Vero cell banks, and reporter virus stock and qualified equipment, and using validated data acquisition and processing systems. The validated assay LOD and LLOQ were 19. For GMT calculations, values below the LOD and LLOQ were assigned a value of 9.5 (half the LOD).

### Euthanasia, tissue collection, and histopathological evaluation

Animals were euthanized with an overdose of sodium pentobarbital, followed by necropsy. Tissues were collected and preserved using three methods depending on the analysis: (1) snap-frozen in liquid nitrogen and stored at ≤–70 °C for quantification of infectious virus by titration; (2) placed in RNAlater® and stored at ≤–20 °C for CHIKV RNA quantification via RT-qPCR; or (3) fixed in 4% paraformaldehyde (PFA) at room temperature for 24 h, then transferred to 70% ethanol for storage until processing. The fixed samples were then paraffin-embedded, sectioned, and used for in situ hybridization (ISH) to detect viral RNA using RNAScope®, and for hematoxylin and eosin (H&E) staining. A second set of tissues was preserved in 10% neutral buffered formalin as backup. Tissues from a second uninfected animal were also used as a control for histological analyses. H&E-stained slides were reviewed by a board-certified pathologist blinded to group assignments. Joint tissues, including adjacent tendons and muscles, were scored based on inflammation severity and the presence of fibrin and/or neutrophils, to allow objective comparisons across study groups. Scores from stifle, ankle, and wrist joints, including capsule/synovium and muscle tendons were combined using a scoring metric (Table 2). The total pathology score of all tissues includes the lymphoid tissues and major organs. Synovial fluid was also collected, stained with Wright-Giemsa, and evaluated cytologically.

**Table 2:** Criteria for grading tissues for histologic evaluations

Joint/synovium		
Inflammation		
	1	Minimal inflammation = Few scattered individual inflammatory cells or aggregates of less than 10
	2	Mild inflammation = Inflammation forms small aggregates of 10–50 cells per aggregate
	3	Moderate inflammation = Inflammatory cells form aggregates of more than 50 cells per aggregate
Fibrin	1	Add additional point for presence of fibrin/necrosis
Neutrophils	1	Add additional point presence of neutrophils

Muscle/tendon		
Inflammation directly affecting muscle or tendon (if between muscle and tendon count towards muscle)		
	1	Minimal inflammation = Few scattered individual inflammatory cells or aggregates of less than 10
	2	Mild inflammation = Inflammation forms small aggregates of 10–50 cells per aggregate
	3	Moderate inflammation = Inflammatory cells form aggregates of more than 50 cells per aggregate
Fibrin	1	Add additional point for necrosis/degeneration
Neutrophils	1	Add additional point presence of neutrophils
Fascia	1	Add additional point for inflammation is surrounding fascia

Lymph nodes/spleen		
Inflammation: Aggregates of neutrophils in lymph node not in sinuses/ peri-follicular aggregates in spleen		
	0	No inflammation
	1	Inflammation present

Follicular Hyperplasia		
	NA	Tissue not present
	0	no follicles in germinal centers
	1	Few scattered germinal centers in follicles
	2	Most follicles have large germinal centers
	3	All follicles have germinal centers

All other tissues (bone, liver, kidney, lung, skin, eyes, CNS)		
Inflammation		
	0	no inflammation or significant lesions related to CHIKV
	1	Inflammation (only included inflammation not considered a background lesion)

#### In situ hybridization using RNAScope

In situ hybridization (ISH) was performed using the RNAscope® platform (Advanced Cell Diagnostics [ACD], Newark, CA, #322310-USM) with minor modifications. Tissue samples were collected within 45 min of the cessation of blood circulation, fixed in 4% paraformaldehyde at room temperature for 24 h, and then stored in 70% ethanol prior to paraffin embedding. Paraffin-embedded tissue sections (4 µm thick) were deparaffinized with xylene and pretreated sequentially with hydrogen peroxide (H₂O₂), RNAscope® target retrieval solution, and RNAscope® Protease Plus under optimized conditions. Sections were hybridized with CHIKV-specific probes (V-CHIKV-O1, ACD) at 40 °C for 2 h, followed by signal amplification steps. Detection was performed using 3,3′-diaminobenzidine (DAB), and slides were counterstained with hematoxylin, dehydrated, cover-slipped, and examined under a bright field microscope. Positive controls included probes for the housekeeping gene peptidylprolyl isomerase B. Negative controls consisted of the same tissues from an uninfected animal and probes targeting the bacterial gene dihydrodipicolinate reductase. Slides were scanned at 20× or 40× magnification without knowledge of treatment group assignment. ISH staining was scored on a 0–4+ scale based on predefined criteria (Table 3), which included the number and distribution of signal dots, presence of dot clusters, and the number of germinal centers showing signal.

**Table 3:** RNAscope In Situ Hybridization (ISH) scoring criteria for tissues

	0	1+	2+	3+	4+
Dots and % of “+” germinal center	No	A few, <25%	Many dots, 25–50%	Many dots, 50–75%	Many dots, >75%
Dot clusters in germinal center	No	No	A few	A lot	Intensive clusters
Dots in mantle zone	No	No	A few	A lot	A lot with clusters
Dots in paracortex, medulla and red pulp	No	No	No	Yes	Yes, with clusters

All other tissues.

0: no staining.

1+: a few dots on slides.

2+: many dots with a few positive cells having dot clusters foci on the slide.

3+: many dots scattered on the slide with a few positive cells having dot clusters.

4+: a lot of dots easily found on the slide, with a lot of positive cells have intensive dot clusters.

### Statistical analyses

Statistical analyses were conducted using Prism 10 (GraphPad, San Diego, CA) or R (version 3.6.0). Data distributions were assessed prior to analysis, and because assumptions of normality and homogeneity of variance were not met, non-parametric statistical tests were used. Comparisons among multiple groups were performed using the Kruskal–Wallis test, followed by pairwise comparisons using the Mann–Whitney U test where appropriate. The specific statistical tests used are indicated in the text and figure legends. Any data transformations are described in the relevant results sections or figure legends. Unless otherwise noted, all *p*-values are two-sided, and *p*-values ≤ 0.05 were considered statistically significant. Where applicable, *p*-values were adjusted for multiple comparisons. CHIKV NT_80_ titers were calculated using the PaxVaxCI4GxP R package for Clinical Immunology (versions 0.7.0 and 0.8.0).

## Results

### CHIKV at doses of 10^6^ to 10^8^ PFU infects cynomolgus macaques to produce similar viremia magnitudes and kinetics

**Fig. 1:**
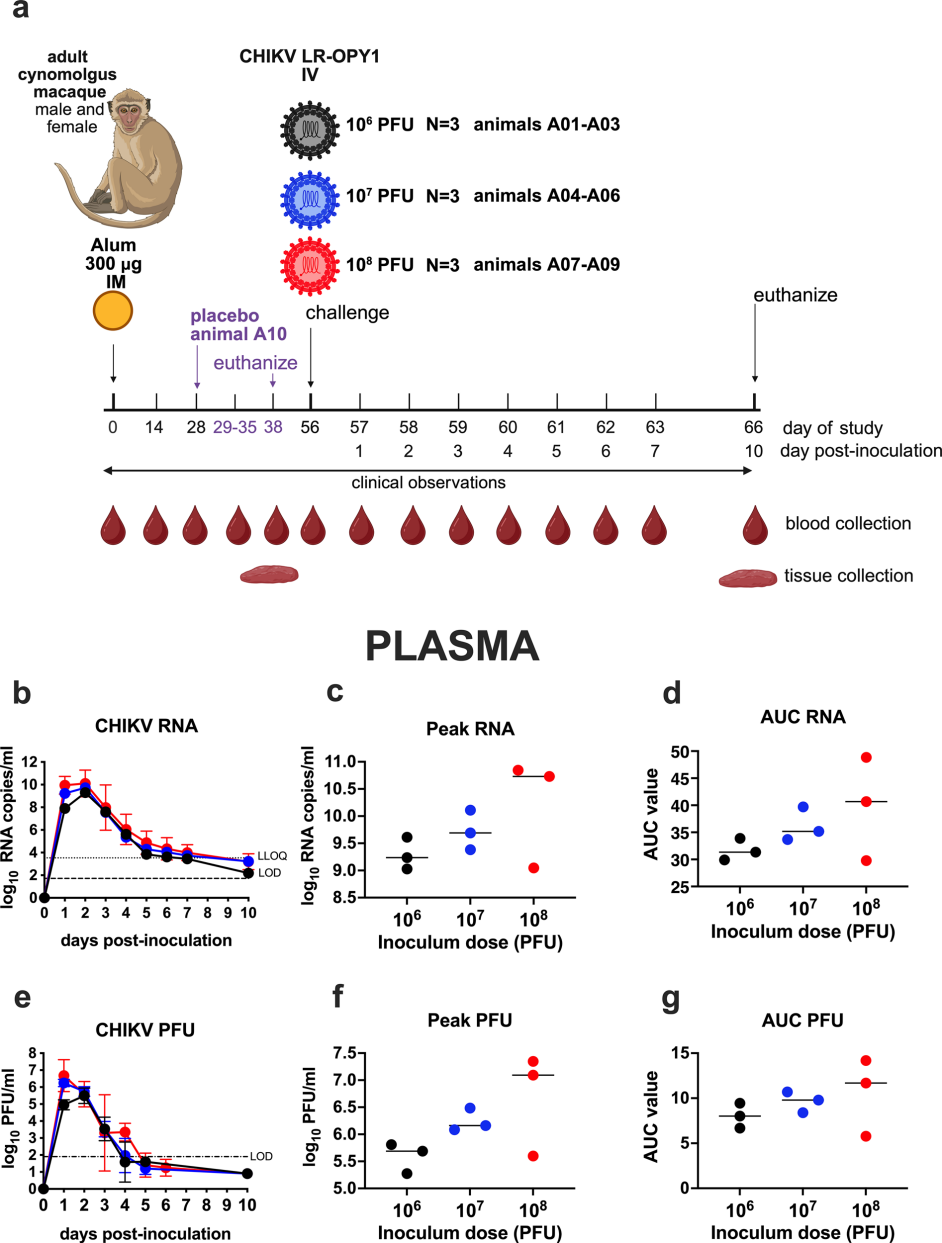
CHIKV plasma viremia kinetics in cynomolgus macaques inoculated with one of 3 different virus doses, Study A. **a** Experimental design. **b** Mean CHIKV RNA levels in plasma over time. **c** Peak CHIKV RNA levels by dose group. **d** Area under the curve (AUC) for CHIKV RNA levels. **e** Mean Infectious CHIKV levels in plasma. **f** Peak infectious CHIKV levels. **g** AUC for infectious CHIKV levels. Data are based on log-transformed values above the LOD. Error bars show standard deviations. Group means are indicated by short lines. Dotted lines show LOD and LLOQ. Statistical significance was assessed using Kruskal–Wallis tests.

Three different challenge doses (10^6^, 10^7^ or 10^8^ PFU) of CHIKV strain LR2006-OPY1 administered intravenously (IV) were evaluated to measure CHIKV RNA and infectious virus levels in blood samples collected daily from baseline day 0 through day 7, and 10 days post-challenge in 9 cynomolgus macaque NHPs (Fig. 1a). All 9 animals developed detectable CHIKV RNA in plasma. The mean levels at the peak, day 2, were 9.2 ± 0.2 (10^6^ group), 9.7 ± 0.3 (10^7^ group), and 10.1 ± 1.2 (10^8^ group) log_10_ RNA copies/ml plasma (Fig. 1b). By day 10 post-challenge, RNA levels declined to the limit of quantitation (LOQ) of the RT-qPCR assay. Animals that received higher CHIKV challenge doses showed slightly higher peak RNA levels in plasma (Fig. 1c) and area-under-the-curve (AUC) values (Fig. 1d); however, these differences among the 3 dose groups were not statistically significant (*p* > 0.05, Kruskal–Wallis test). Infectious virus levels in plasma, measured in plaque forming units per milliliter (PFU/ml), followed a similar pattern, with mean levels at peak of 5.5 ± 0.2 (day 2, 10^6^ group), 6.2 ± 0.2 (day 1, 10^7^ group), and 6.7 ± 0.8 (day 1, 10^8^ group) (Fig. 1e). As with RNA, peak PFU titers (Fig. 1f) and AUC values (Fig. 1g) tended to increase with dose but did not differ significantly among groups (*p* > 0.05, Kruskal–Wallis test). These findings indicated that viremia kinetics and magnitude are largely comparable across the 100-fold CHIKV dose range from 10^6^ to 10^8^ PFU administered IV to cynomolgus macaques. All but one of the back-titrated CHIKV inocula were within the expected 10-fold range of the target dose (Supplementary Fig. 1). CHIKV plasma viremia kinetics on a per animal level are shown in Supplementary Fig. 2.

As expected, no animals were found deceased or were euthanized other than at the scheduled study endpoints. Moribund CHIKV-infected cynomolgus macaques displayed signs consistent with CHIK disease including lymphopenia, elevated markers of liver and muscle function, histopathologic signs of joint inflammation with CHIKV RNA in lymphoid tissues and muscles and necessitated supportive care. Animals inoculated with CHIKV showed fluctuations in rectal temperature (Supplementary Fig. 3) and received analgesic treatment (Supplementary Fig. 4) based on clinical signs of pain, administered by CNPRC veterinarians blinded to groups. The 10⁸ PFU group began supportive care earlier and required it longer, though not significantly (*p* > 0.05, Logrank test). Supportive care included subcutaneous fluids and, in some cases, orogastric high-calorie formula, which likely helped maintain stable body weights despite clinical disease. By study end, animals stabilized and required only analgesics; no euthanasia criteria were met before the 10-day endpoint. The placebo animal required no supportive care. CHIKV-infected macaques showed transient lymphopenia within two days, with lymphocyte counts dropping 2.2- to 6-fold from baseline. Neutrophil and monocyte counts were variable in all animals (Supplementary Fig. 5). Alanine transaminase (ALT) and aspartate transferase (AST) rose transiently in all CHIKV-infected animals, especially in the 10⁸ PFU group, and C-reactive protein (CRP) was elevated in most animals (Supplementary Fig. 6). Necropsies performed 10 days post-inoculation revealed no gross abnormalities. Histological analysis focused on joints and adjacent muscles, with lesions scored for inflammation, fibrin, necrosis/degeneration, and neutrophils (Table 2, Supplementary Fig. 7). All CHIKV-infected animals showed joint inflammation in stifle, wrist, and ankle joints (Supplemental Fig. 8), whereas the placebo control had no lesions. Combined together, the CHIKV-infected animals had significantly higher ankle, wrist, and overall pathology scores (*p* ≈ 0.03, Kruskal–Wallis). No brain inflammation was observed. CHIKV RNA, assessed by RNAScope ISH, persisted in lymphoid and muscle tissues, including inguinal lymph node, spleen, liver, synovium, and muscle, with strongest signals in spleen, lymph nodes, and muscle (Supplementary Fig. 9). Animals receiving 10⁸ PFU had higher RNAScope scores compared to the 10^6^ PFU group (*p* = 0.017, Kruskal–Wallis, Dunn’s Multiple comparisons test). Based on analysis of this viral, clinical, and histopathological data from Study A demonstrating similarities to human CHIKV disease at all evaluated doses, while also considering the need to avoid excessive inflammation, immune activation, and mortality, the intermediate dose of 10^7^ PFU CHIKV was selected for use in Studies B and C.

### A CHIKV VLP vaccine reduces CHIKV viremia in cynomolgus macaques

**Fig. 2:**
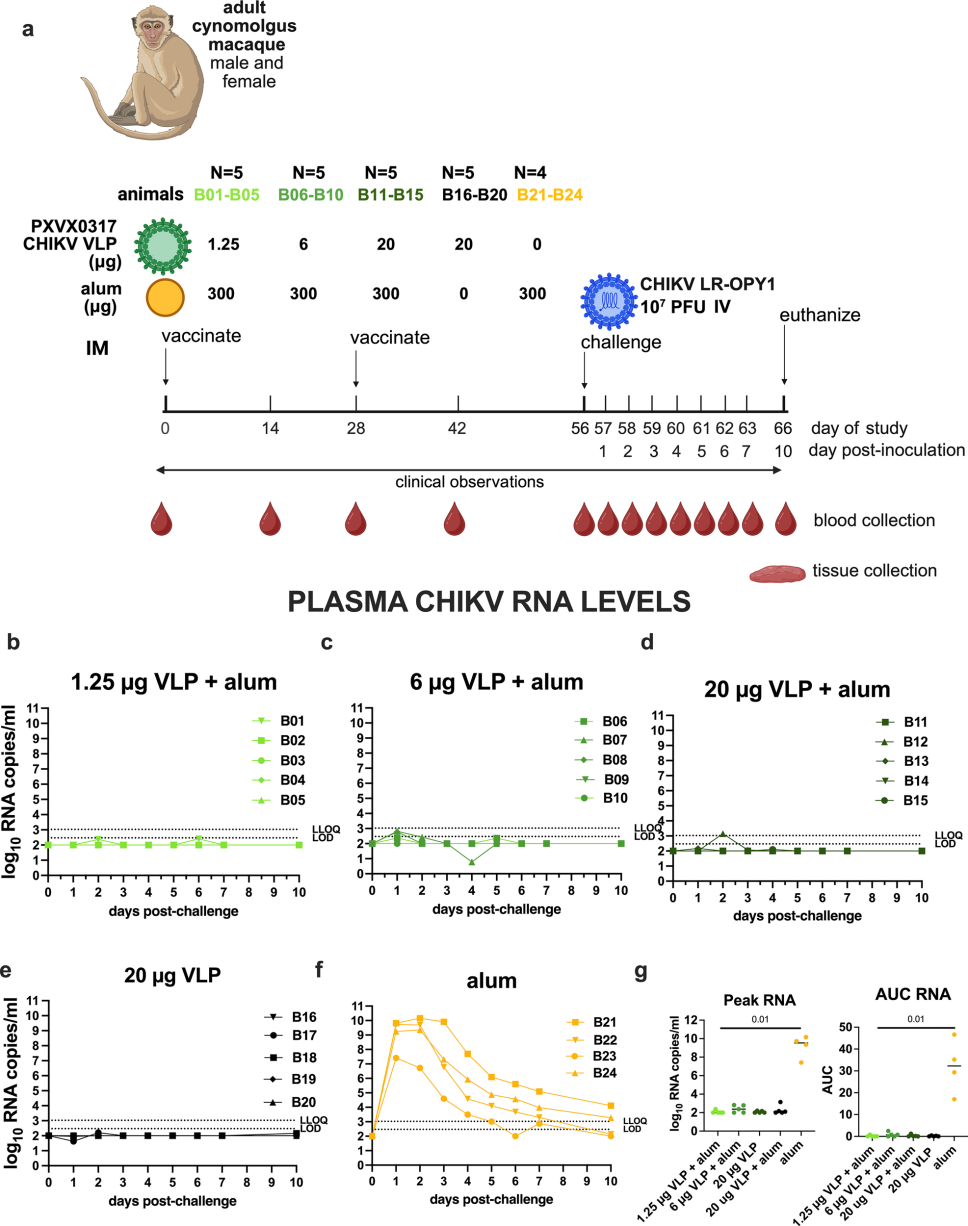
CHIKV plasma viremia kinetics in cynomolgus macaques vaccinated with one of 3 doses of CHIKV VLP + aluminum hydroxide (alum), or VLP or aluminum hydroxide only and then challenged with 10^7^ PFU CHIKV LR-OPY1, Study B. **a** Experimental design. **b**–**f** CHIKV RNA levels in plasma, and **g** (left) peak and (right) AUC of CHIKV RNA. Each line shows measurements from one animal. AUC was calculated on log-transformed RNA levels using only the area above the LOD. Group means are indicated by short lines in panel **g**. The LOD and LLOQ are indicated by horizontal dotted lines. *P*-values are based on Mann–Whitney tests.

**Fig. 3:**
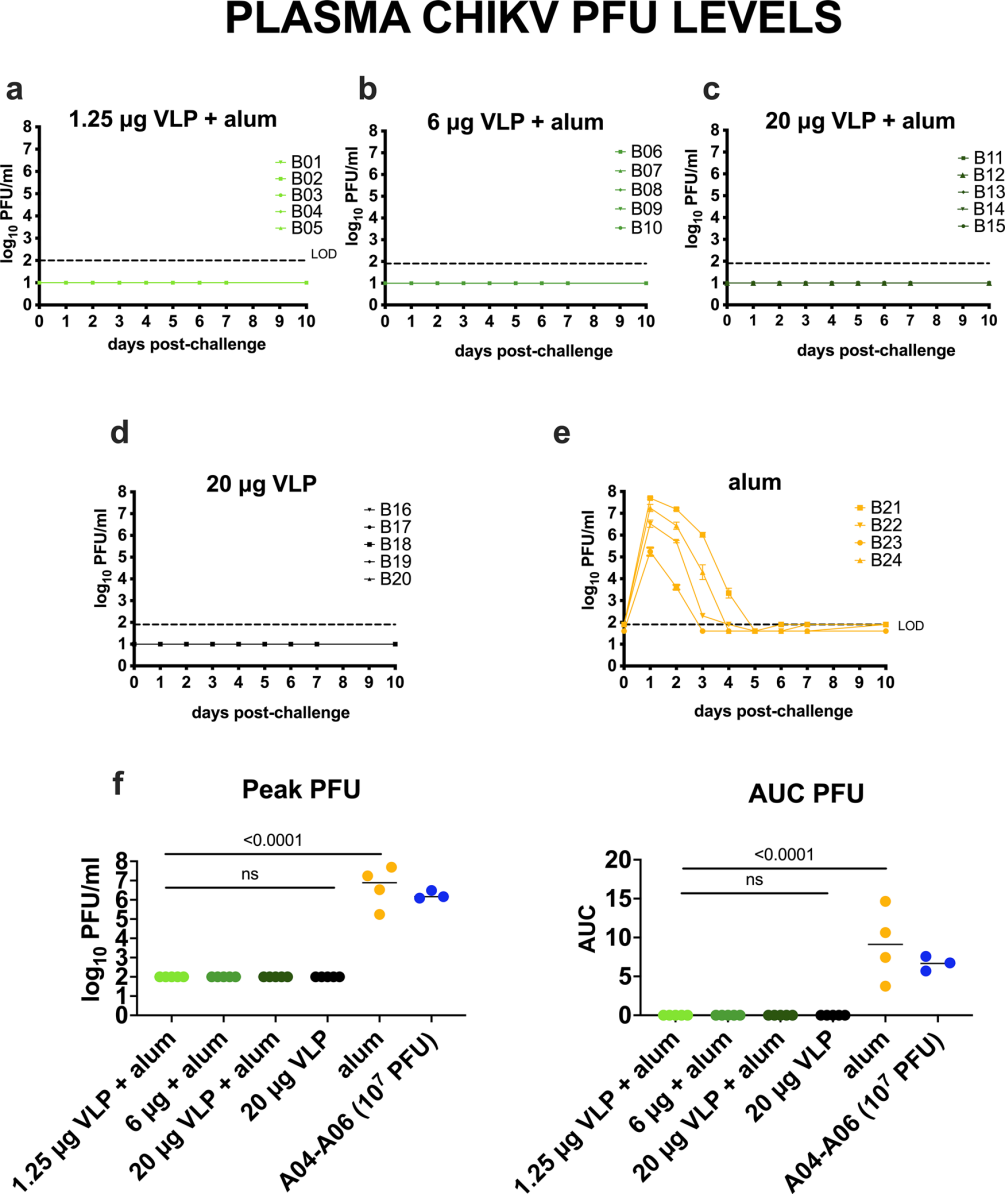
Infectious CHIKV levels in plasma measured in plaque forming units for CHIKV VLP vaccinated or aluminum hydroxide (alum) administered and CHIKV challenged cynomolgus macaques, Study B. Each line in **a**–**e** shows measurements from one animal. The LOD is indicated by the dotted line. Samples with no detectable infectious CHIKV were given a value of 1 log_10_ PFU/ml for visualization. AUC analysis of CHIKV PFU over time was based on log-transformed levels using the area above the LOD. Group means are indicated by short lines in panel **f**. The 3 animals from Study A (A04-A06) that received the same dose (10^7^ PFU) of CHIKV LR-OPY1 are shown for comparison.

The goal of Study B was to evaluate the immunogenicity and efficacy of the CHIKV VLP vaccine in cynomolgus macaques, formulated with or without aluminum hydroxide (Fig. 2a). The vaccine was well tolerated, with no adverse effects observed. Following challenge, all four CHIKV VLP-vaccinated groups had minimal plasma CHIKV RNA (Fig. 2b–e) with 11 of 20 animals being undetectable (e.g. below the LOD), and the remaining 9 animals showing levels between the LOD and LLOQ at 1-, 2-, 5-, or 6-days post challenge. Only one animal (B12, 20 μg VLP+ aluminum hydroxide) exceeded the LLOQ on day 2. In contrast, adjuvant-only control animals developed high viremia, peaking at 7.4–10.2 log_10_ RNA copies/ml plasma within 1–2 days post-challenge (Fig. 2f). Infectious virus kinetics mirrored CHIKV RNA results; no infectious virus was detected in vaccinated animals (Fig. 3a–d) but adjuvant-only animals showed detectable virus (5.2–7.7 log_10_ PFU/ml) that cleared by days 3–5 (Fig. 3f). Vaccine groups had significantly reduced RNA and infectious virus levels compared to controls, both for peak RNA and PFU levels and AUC (Figs. 2g and 3f), and combined vaccine groups showed a significant reduction in detectable CHIKV RNA compared to the adjuvant only group (*p* < 0.0001; Mann–Whitney test). Two doses of CHIKV VLP, given on days 0 and 28, effectively suppressed both RNA and infectious virus following challenge on day 56. Because 20 µg unadjuvanted VLP achieved similar suppression to alum-adjuvanted groups, any additional benefit of aluminum hydroxide at this dose could not be determined. Back-titration showed that most inocula were within the expected 10-fold range of the target titer (1.92 ×10^7^ PFU/ml), though several were slightly above it (Supplementary Fig. 10).

### A CHIKV VLP vaccine reduces clinical disease in cynomolgus macaques

Fevers ≥103 °F (99-102 °F is the normal range) were observed in two aluminum hydroxide-only animals and one 20 μg VLP + aluminum hydroxide animal (Supplementary Fig. 11); all were treated with acetaminophen. Weight remained stable across groups, aided by nutritional supplements and orogastric intubation. The aluminum hydroxide-only group required the most supportive care, including buprenorphine and ketoprofen, while higher-dose CHIKV VLP groups needed minimal analgesics (Supplementary Fig. 12). White blood cell and monocyte counts varied without clear treatment trends (Supplementary Fig. 13), though transient lymphopenia occurred in most animals, most pronounced in the aluminum hydroxide-only group (*p* = 0.006, ANOVA; Supplementary Fig. 14). Biomarkers of liver and muscle injury, ALT, AST, CRP, showed intra-animal variability across all groups (Supplementary Fig. 15).

### CHIKV VLP vaccine induces CHIKV-specific neutralizing antibody responses

**Fig. 4:**
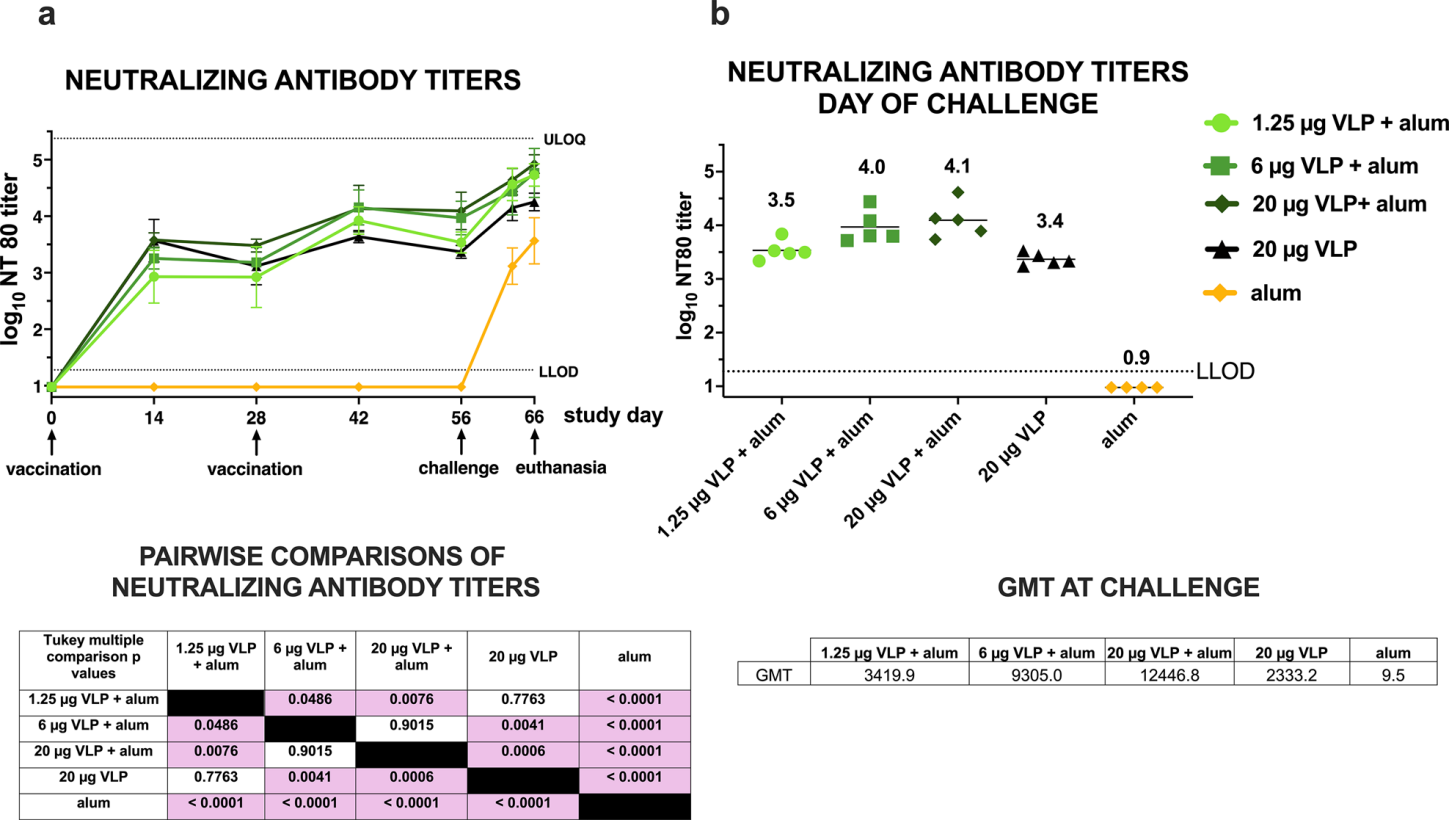
Temporal kinetics of CHIKV neutralizing antibody titers after CHIKV VLP vaccination or aluminum hydroxide (alum) treatment and CHIKV challenge in cynomolgus macaques. **a** Kinetic changes in mean neutralization antibody titers (NT_80_). Each line represents the cohort mean and error bars show standard deviations. Pairwise comparisons of day 56 pre-challenge titers between groups used Tukey’s multiple comparisons and pink shading highlights *p*-values < 0.05. **b** NT_80_ titers on the day of CHIKV challenge, study day 56. Each dot represents an individual; horizonal lines indicate group means, also shown above groups. The accompanying table shows geometric mean titers (GMTs) on the day of challenge. For calculating GMTs, NT_80_ titers below the LOD of 19 were assigned a value one half the LOD (9.5) and values greater than the assay upper limit of quantification (ULOQ) were assigned a value of the ULOQ. LLOD is the lower limit of detection.

Mean neutralizing antibody titers increased across all vaccine groups from study day 0 to 56, the day of CHIKV challenge (Fig. 4a). All animals vaccinated with the CHIKV VLP — whether adjuvanted with aluminum hydroxide or not — developed detectable neutralizing antibody responses (Supplementary Fig. 16). Mean neutralizing titers on day 56 (28 days following the second dose) were 3.4 ± 0.1 (20 μg VLP only), 3.5 ± 0.2 (1.25 μg VLP+ aluminum hydroxide), 4.0 ± 0.3 (6 μg VLP+ aluminum hydroxide), and 4.1 ± 0.3 (20 μg VLP+ aluminum hydroxide) log_10_ (Fig. 4b). In contrast, the alum-only group had no detectable neutralizing titers above the LLOD prior to CHIKV challenge. The geometric mean titer (GMT) on the day of challenge differed significantly between the five groups (*p* < 0.0001, one-way ANOVA) (Fig. 4, right table). Tukey multiple comparison tests (Fig. 4, left table) revealed that GMTs were significantly lower in the aluminum hydroxide-only group compared to any CHIKV VLP dose + aluminum hydroxide group. Titers in the 6 μg VLP + aluminum hydroxide and 20 μg VLP + aluminum hydroxide groups were higher than in the 20 μg VLP and 1.25 μg VLP + aluminum hydroxide groups. Following CHIKV challenge, titers in 19 of 20 immunized animals increased by 0.6 to 1.5 log_10_ between study days 56 and 66, 10 days after CHIKV challenge. The aluminum hydroxide-only group also developed a primary neutralizing antibody response, reaching titers of 3 to 4 log_10_ by study day 66, the study endpoint. These findings demonstrate that the CHIKV VLP vaccine is immunogenic, inducing neutralizing antibody responses that scale with VLP dose for adjuvanted formulations. Moreover, addition of adjuvant increased immunogenicity, as animals immunized with 1.25 μg VLP+ aluminum hydroxide achieved similar titers to those receiving 20 μg VLP without aluminum hydroxide.

### CHIKV VLP vaccine reduces CHIKV RNA levels in tissues and histopathologic signs of inflammation in joints

**Fig. 5:**
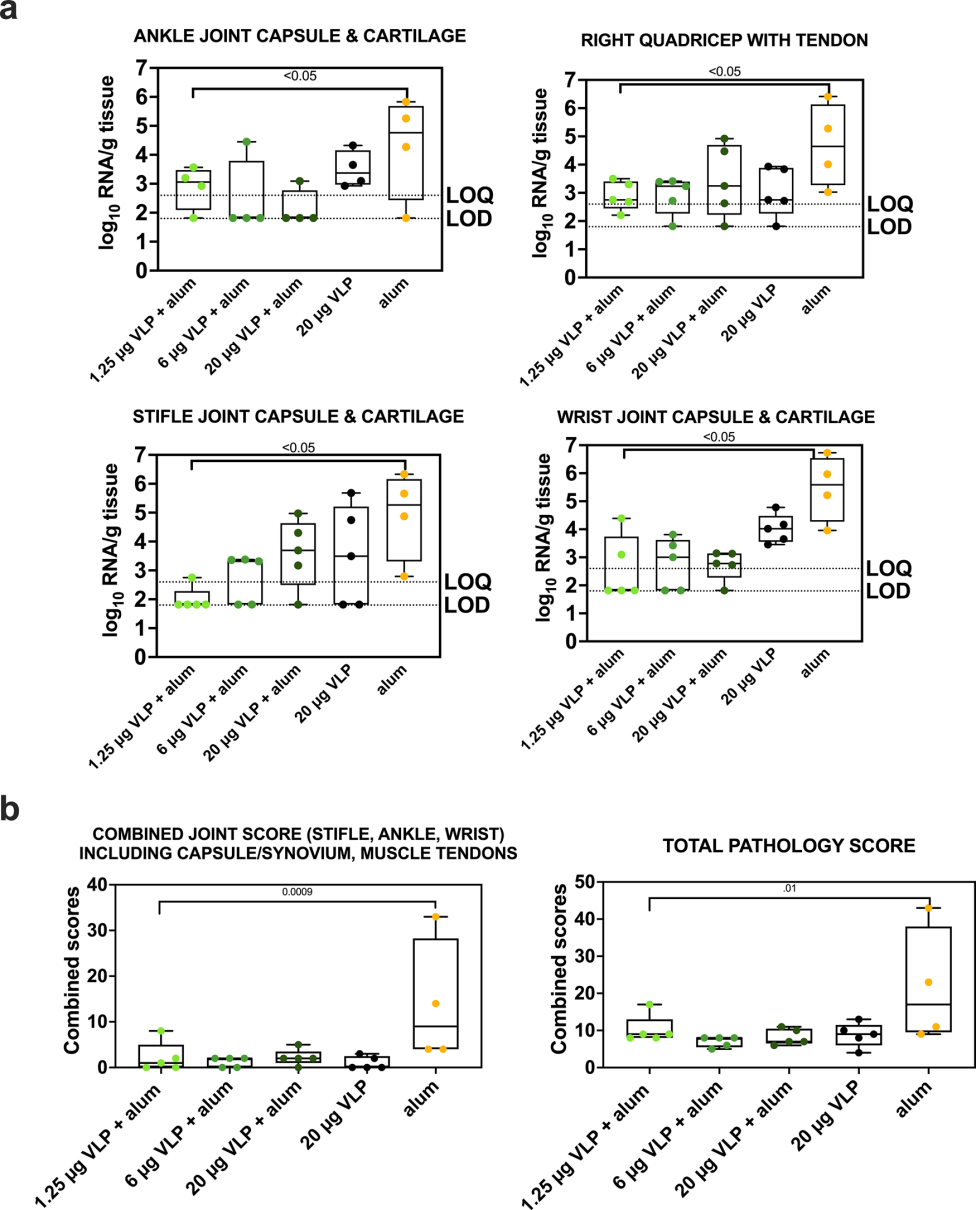
CHIKV RNA levels and histopathology scores in joint tissues and muscles and tendons after CHIKV VLP vaccination or aluminum hydroxide (alum) treatment and CHIKV challenge in cynomolgus macaques. **a** CHIKV RNA levels in ankle joint capsule and cartilage, right quadricep with tendon, stifle joint capsule and cartilage, and wrist joint capsule and cartilage. **b** Histopathology scores in joints and all tissues. For all panels, each dot represents one animal, box plot lines indicate group means, and whiskers indicate the full range. Samples with undetectable CHIKV RNA were assigned a value at the LOD. *P* values are from Tukey–Kramer and Mann–Whitney tests.

All animals had detectable CHIKV RNA in most of the 19 evaluated tissues 10 days post-challenge. Animals in the aluminum hydroxide-only group showed higher CHIKV RNA levels across most tissues, similar to levels observed in animals from Study A that received 10^7^ PFU (Supplementary Fig. 17). In joint-associated tissues, including joints, muscles, and tendons, CHIKV RNA levels per gram of tissue were significantly higher (*p* < 0.01; Tukey–Kramer test) in untreated and aluminum hydroxide-only animals compared to CHIKV VLP-vaccinated animals. No significant differences in CHIKV RNA levels were observed among the different vaccine doses or formulations (*p* > 0.05, Tukey–Kramer test). RNAscope scores in animals that received any CHIKV VLP dose were also significantly lower in lymphoid tissues, liver, heart, and spinal cord compared to animals that received aluminum hydroxide only (*p* < 0.0001, Kruskal–Wallis test) (Supplementary Fig. 18). Animals in the aluminum hydroxide-only group had significantly higher pathology scores in the ankle (*p* < 0.05), stifle (*p* < 0.05), wrist joints (*p* < 0.05), quadricep muscles (*p* < 0.05), combined joint scores (*p* = 0.0009) and overall tissue scores (*p* < 0.01) compared to all CHIKV VLP groups together (Mann–Whitney test) (Fig. 5). In contrast, pathology scores were low across all CHIKV VLP groups, with no significant differences observed between any CHIKV VLP-treated group. These findings indicate that the CHIKV VLP vaccine reduces CHIKV RNA levels in tissues and histologic evidence of joint and overall tissue inflammation 10 days following CHIKV infection.

Collectively, the data from Study B show that CHIKV VLP-vaccinated animals had lower plasma and tissue CHIKV RNA levels, no detectable infectious viremia, fewer clinical signs and reduced need for supportive care, and lessened joint and muscle pathology. All vaccinated animals also developed high neutralizing antibody titers, further supporting immunogenicity of the CHIKV VLP vaccine.

### Passive transfer of IgG from CHIKV VLP-vaccinated humans reduces CHIKV viremia in cynomolgus macaques

**Fig. 6:**
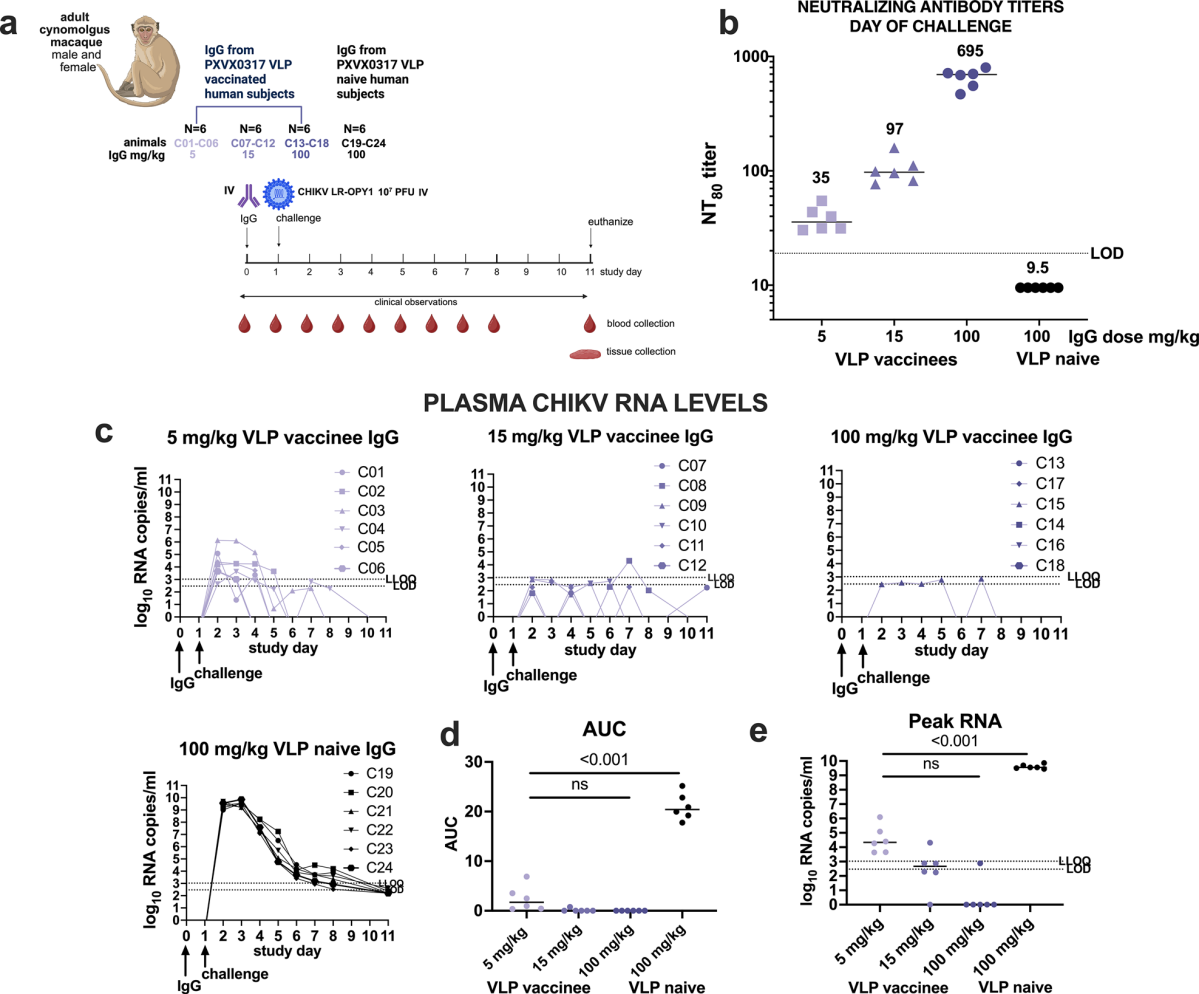
CHIKV plasma viremia kinetics in cynomolgus macaques administered IgG from CHIKV VLP vaccinated or naive people and then challenged with 10^7^ PFU CHIKV LR-OPY1, Study C. **a** Experimental design, **b** Neutralizing antibody titers on the day of challenge 1 day after passive transfer of IgG, **c** CHIKV RNA levels in plasma, **d** AUC analysis of CHIKV RNA over time and, **e** Peak CHIKV RNA levels. Each line in (**c**) shows measurements from one animal. The LOD and LLOQ are indicated by horizontal dotted lines. AUC was calculated on log-transformed RNA levels using only the area above the LOD of the RT-PCR assay.

**Fig. 7:**
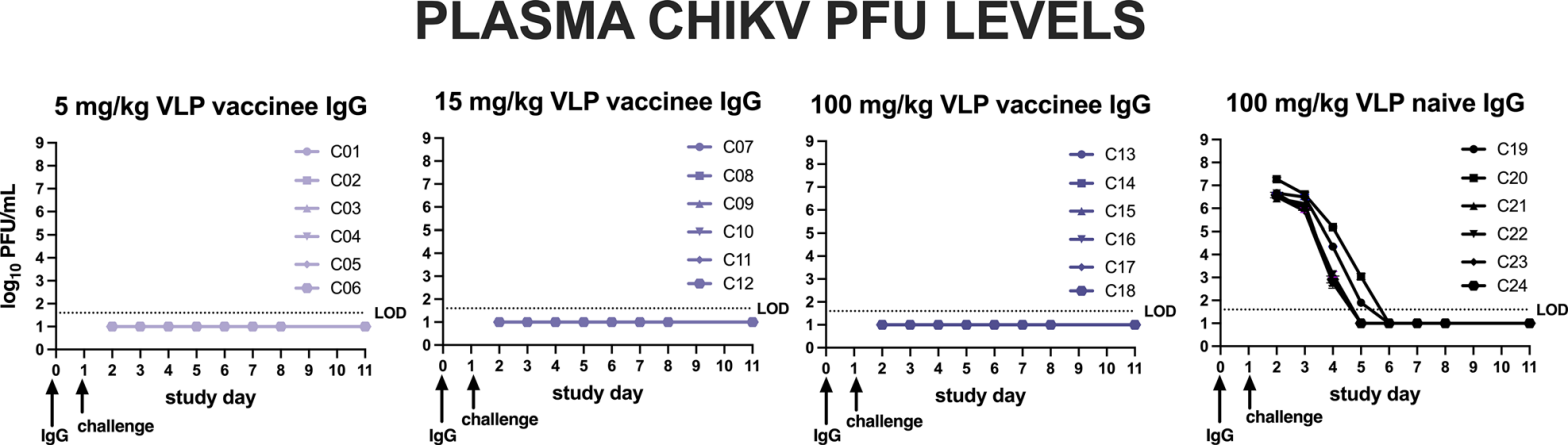
Infectious CHIKV levels in plasma measured in plaque forming units for animals that received antibody from CHIKV VLP vaccinated or naïve people. Each line shows measurements from one animal. The LOD is indicated by the line at 1.7 log_10_ PFU/mL. Samples with no detectable infectious CHIKV were given a value 1 log_10_ PFU/ml.

In Study C, purified IgG from CHIKV seronegative or CHIKV VLP-vaccinated humans was administered to macaques 1 day prior to IV challenge with 10⁷ PFU CHIKV LR2006-OPY1 (Fig. 6a). On the day of challenge, all animals that received vaccine-induced IgG had detectable NT_80_ titers, with a mean of 35 for animals that received 5 mg/kg, 97 for those receiving 25 mg/kg, and 695 for the 100 mg/kg group, while NT_80_ titers were undetectable in the control group (Fig. 6b). Animals receiving IgG from CHIKV VLP vaccinees had significantly lower CHIKV RNA levels in plasma from 1 to 8 days post-challenge relative to the control group that received IgG from VLP naïve people (Fig. 6c). Protection increased with increasing IgG dose: 5 of 6 animals in the 100 mg/kg group, 1 of 6 in the 15 mg/kg group, and none in the 5 mg/kg group had RNA levels below the LOD. Twelve treated animals had some time points with detectable CHIKV RNA in plasma that ranged from 3 to 6 log_10_ RNA copies/ml, with one of the animals in the 15 mg/kg group and all six animals in the 5 mg/kg treatment group having levels above the LOD. In contrast, all animals that received IgG from VLP naïve people had high plasma CHIKV RNA levels, peaking at 9.4–9.8 log_10_ RNA copies/ml 1–2 days post-inoculation, similar to results from control animals in Studies A and B. The AUC (Fig. 6d) and peak RNA levels (Fig. 6e) were significantly lower (*p* < 0.001, Kruskal–Wallis test) in all groups receiving IgG from CHIKV VLP vaccinees compared to the CHIKV VLP naïve control group. VLP vaccinated groups were not different from each other (*p* > 0.05, Kruskal–Wallis test). Infectious CHIKV was not detected in plasma from any CHIKV VLP IgG–treated animal (LOD: 1.6 log_10_ PFU/ml), while all control animals had detectable infectious virus (peak 6.4–7.3 log_10_ PFU/ml at day 1) that became undetectable by day 6 (Fig. 7). This viremia pattern in controls matched that observed in the 10^7^ PFU Study A group and the adjuvant-only group in Study B. After back titration of inocula, we observed that all but one animal (C01) received doses within the expected 10-fold target range (Supplementary Fig. 19). Animal C01 received a mean inoculum dose of 7.6 log_10_ PFU.

### Passive transfer of IgG from CHIKV VLP-vaccinated humans reduces clinical disease in CHIKV-infected cynomolgus macaques

Mild rectal temperature elevations (102–103 °F) were observed in many animals across groups (Supplementary Fig. 20) and were likely due to agitation before sedation. After CHIKV inoculation, 2 animals (C20 and C21) in the control group that received IgG from CHIKV VLP-naïve humans, and one animal (C08) in the 15 mg/kg vaccinee IgG group, developed an elevated rectal temperature ≥103 °F and were treated with ketoprofen. No other animals reached this temperature threshold or required treatment. Body weights generally remained stable throughout the study, likely due to consistent nutritional support and orogastric intubation. Animals that received IgG from VLP-naïve humans required the most supportive care, including the highest number of long-acting buprenorphine (*n* = 11) and ketoprofen (*n* = 40) doses (Supplementary Fig. 21) and the greatest number of days with fluid or nutritional supplementation (*n* = 25). In contrast, animals that received IgG from VLP vaccinees also required some supportive care, but the number of treatments with buprenorphine and ketoprofen was generally lower than in the control group. By the end of the study, all animals were clinically stable and no longer required additional supportive care.

### Passive transfer of IgG from CHIKV VLP-vaccinated humans reduces tissue CHIKV RNA levels and histopathologic signs of inflammation in joints

**Fig. 8:**
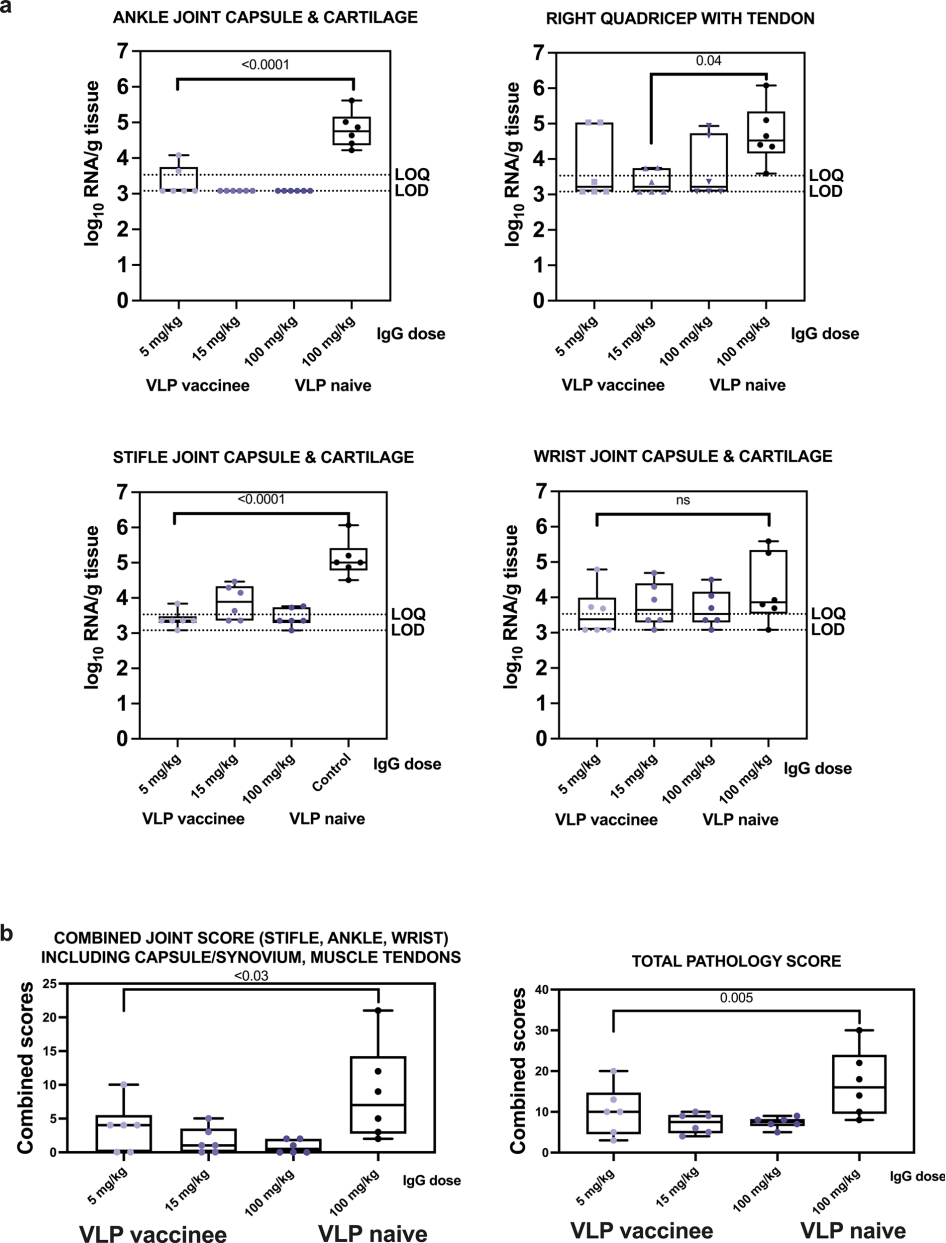
CHIKV RNA levels and histopathology scores in joint tissues and muscles and tendons after CHIKV IgG administration and CHIKV challenge in cynomolgus macaques. **a** CHIKV RNA levels in ankle joint capsule and cartilage, right quadricep with tendon, stifle joint capsule and cartilage, and wrist joint capsule and cartilage and **b** Histopathology scores in joints and all tissues. Each dot shows the value from one animal, lines in boxes show means, and whiskers show ranges. Samples with undetectable CHIKV RNA were assigned a value at the LOD for graphing and statistical analysis. Statistical designations are based on ANOVA with Tukey’s multiple comparisons tests.

Animals that received IgG from VLP-naïve humans had significantly higher levels (*p* < 0.001, ANOVA) of CHIKV RNA across all tissues compared to those administered IgG from VLP vaccinees (Supplementary Fig. 22). Among the groups administered different doses of IgG from vaccinated humans, there were no statistically significant differences in tissue RNA levels (*p* > 0.05, ANOVA). While most animals had detectable CHIKV RNA in most tissues (Supplementary Fig. 23), the viral RNA levels were consistently lower in animals that received CHIKV VLP-vaccinee IgG, with the greatest reductions observed in lymph nodes. RNAscope scores in animals that received any IgG dose from VLP-vaccinees were also significantly lower in lymphoid tissues and liver compared to animals that received IgG from VLP-naïve people (*p* = 0.002, Kruskal–Wallis test) (Supplementary Fig. 24). Animals administered IgG from VLP-naïve humans had significantly higher inflammation scores in the ankle joint (*p* = 0.0001), quadriceps muscle (*p* = 0.04), stifle joint (*p* = 0.0001), combined joint score (*p* < 0.03), and overall pathology across all tissues (*p* = 0.005) (Mann–Whitney tests) compared to animals that received IgG from VLP-vaccinees (Fig. 8). There were no significant differences between the different IgG from VLP-vaccinees dose groups (*p* > 0.05, Mann–Whitney). These results demonstrate that passively transferred IgG from CHIKV VLP-vaccinated humans effectively reduces CHIKV RNA levels, histopathologic signs of joint inflammation, and overall tissue damage following CHIKV infection of cynomolgus macaques.

### Correlates of protection

**Fig. 9:**
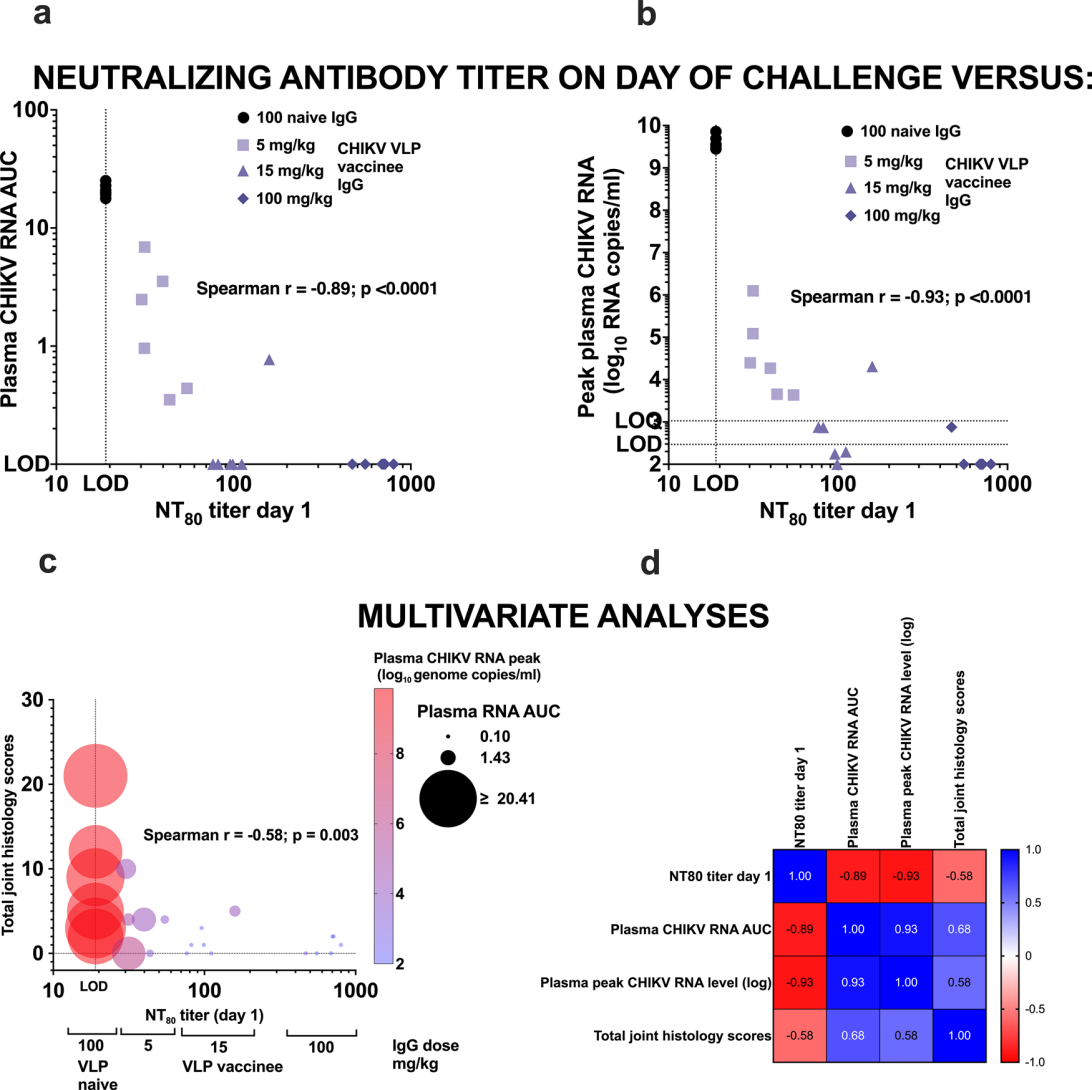
Correlation between serum neutralizing antibody and plasma viral RNA levels. NT_80_ titer shows neutralizing antibody levels on day 1 (the day of CHIKV challenge) correlated with (**a**) plasma CHIKV RNA AUC and (**b**) peak viremia. Multivariate analysis. **c** Bubble plot showing neutralizing antibody titers on day 1 (just prior to CHIKV challenge), total joint histology scores, and peakand AUC values of CHIKV RNA levels in plasma, (**d**) Spearman r multiple variable correlation matrix.

Since passively transferred IgG from VLP-vaccinees effectively reduced CHIKV RNA levels in plasma and tissues in a dose-dependent manner, validating antibody as an immune correlate of protection, we next calculated the level of neutralizing antibody responses associated with efficacy. We analyzed the relationship between serum neutralizing antibody titers on the day of CHIKV challenge and the likelihood of detecting plasma CHIKV RNA above the LLOQ or LOD of the RT-qPCR assay. Serum neutralizing titers were inversely correlated with both AUC (Fig. 9a) and peak CHIKV RNA levels (Fig. 9b) in plasma, and these associations were highly significant (*p* < 0.0001, Spearman test). A multivariate analysis was also conducted to evaluate the relationships among serum neutralizing antibody titers on day 1, peak and AUC plasma CHIKV RNA levels, and total joint histopathology scores (Fig. 9c). There was a strong inverse correlation between day 1 neutralizing antibody titers and both peak and AUC CHIKV RNA levels in plasma. Additionally, total joint histology scores showed a moderately strong inverse correlation with day 1 neutralizing antibody titers as well as moderately strong positive correlations with peak and AUC RNA levels. All correlations were statistically significant (*p* ≤0.003; Fig. 9d).

## Discussion

These preclinical studies were designed and performed to support the clinical development of the CHIKV VLP vaccine at a time when human trials demonstrated safety and immunogenicity, but when performing randomized placebo-controlled efficacy trials faces many logistical hurdles due to the unpredictability of CHIKV outbreaks in many endemic areas. In such situations, animal models can help bridge this gap by providing valuable insights on the efficacy and correlates of vaccine protection to supplement data from human trials. We used a highly relevant cynomolgus macaque model that reproduces common manifestations of viremia and arthritis in CHIKV-infected humans to evaluate the protective efficacy of a West African-lineage CHIKV VLP vaccine and human IgG from CHIKV VLP vaccinees following ECSA-lineage CHIKV challenge. In Study A, we tested three different IV challenge doses (10⁶, 10⁷, and 10⁸ PFU) of ECSA-lineage CHIKV strain LR2006-OPY1 to assess clinical disease and viremia magnitude and kinetics, ultimately selecting a challenge dose of 10⁷ PFU for use in later studies. Study B evaluated the immunogenicity and efficacy of CHIKV VLP, with and without aluminum hydroxide adjuvant. Building on Study B and the observed correlation between neutralizing antibody titers and protection, we assessed whether IgG from CHIKV VLP vaccinated humans could protect macaques from CHIK disease in Study C. In Study A, all animals developed high levels of plasma viral RNA and infectious virus, peaking within 2 days post-inoculation and decreasing to low or undetectable levels by day 10, similar to CHIKV infected humans^42^. Clinical signs consistent with pain and discomfort were observed, and all animals showed joint pathology at necropsy. While differences between inoculation doses were not statistically significant, higher doses were associated with greater viremia, more severe clinical signs, increased need for supportive care, and higher joint pathology scores. CHIKV infection was also linked to elevated liver enzymes, muscle enzymes, and C-reactive protein (CRP). Although no animals in the 10^8^ PFU group met euthanasia criteria in Study A, other studies at similar doses noted this possibility^38^, so to minimize the risk of severe disease that could result in unplanned mortality or animals meeting criteria for euthanasia, an inoculation dose of 10^7^ PFU was selected for Studies B and C to balance pathogenicity and safety.

In Study B, animals were immunized intramuscularly on days 0 and 28 with CHIKV VLP (1.25, 6, or 20 μg) plus aluminum hydroxide (300 μg), or VLP (20 μg) alone, or aluminum hydroxide (300 μg) alone. On approximately day 56, animals were challenged intravenously with 10⁷ PFU of CHIKV strain LR2006-OPY1. All VLP immunized animals developed high neutralizing antibody titers (range: 1726–41,174) by the time of challenge. In Study C, designed to more closely bridge human trials with the animal model, cynomolgus macaques received human IgG (5, 15, or 100 mg/kg) purified from CHIKV VLP vaccinees or control IgG (100 mg/kg) from VLP naïve donors on day 0. Animals were challenged the following day with the same CHIKV dose and route as in Study B (10^7^ PFU, IV). Following challenge, all control animals—either alum-only (Study B) or VLP-naïve IgG (Study C)—developed high plasma CHIKV RNA and infectious virus levels, peaking within 2 days and declining by day 10. These animals exhibited clinical signs requiring supportive care and had the expected joint pathology and high viral RNA levels in joint and muscle tissues at euthanasia. In contrast, animals immunized with CHIKV VLP or treated with IgG from CHIKV VLP vaccinees had fewer clinical symptoms, lower levels of liver and muscle enzyme abnormalities and CRP, no detectable infectious viremia, and significantly reduced CHIKV RNA levels in plasma and tissues. At necropsy, these animals had minimal or no joint or muscle pathology. Altogether, these findings in healthy adult male and female cynomolgus macaques support the immunogenicity and protective efficacy of the CHIKV VLP vaccine and demonstrate that neutralizing antibody provides protection from CHIK disease after challenge with heterologous lineage CHIKV. Our findings extend knowledge of immunogenicity and efficacy of the CHIKV VLP beyond earlier studies^24^ by demonstrating that vaccination reduces clinical signs of arthritic disease in addition to viral and viral RNA burdens, by defining the effects of adjuvanted formulations, and by directly testing the functional efficacy of vaccine-induced antibodies from clinical trial participants in a challenge model with a dose that reproduces CHIK disease including joint effusion.

Because prelicensure randomized controlled efficacy trials in CHIKV-endemic regions are challenging and time-consuming, vaccine regulators have adopted serologic markers as surrogates of protection for CHIKV vaccines^43^, although in vitro neutralizing antibody titers are not always predictive of protective immunity against arboviruses (e.g. dengue virus^44,45^). All CHIKV VLP immunized animals in our studies developed CHIKV neutralizing antibody responses. Furthermore, following CHIKV challenge, all CHIKV VLP immunized animals mounted an anamnestic antibody response, reflected as increasing NT_80_ titers 10 days after CHIKV challenge. A micro plaque reduction neutralization test (PRNT) 50% (μPRNT_50_) titer of ≥150 has been established as the threshold needed to protect cynomolgus macaques passively transferred serum from people vaccinated with the other recently licensed CHIKV vaccine (IXCHIQ) from viremia, fever, and hematologic changes^46^. An 80% neutralization titer of ≥100 was established as a surrogate endpoint to support licensure of PXVX0317 based on studies complementary to this project which show that passive transfer of human serum from vaccinees that received CHIKV VLP 40 μg+ aluminum hydroxide in a single dose protects against viremia in NHP^47^. This threshold is higher than PRNT 80% antibody levels ≥1:10 in people previously infected with CHIKV in the Philippines who did not develop symptomatic CHIK disease despite CHIKV activity in the area^46,47^. In Study B, animals vaccinated twice with any CHIKV VLP dose had NT_80_ titers above 100 at the time of challenge. In Study C, animals that received 100 mg/kg IgG from CHIKV VLP vaccinated humans had a median NT_80_ titer of 695 on the day of challenge. The 15 mg/kg and 5 mg/kg groups had lower median NT_80_ titers of 97 and 35, respectively. Despite these lower titers, animals in both lower-dose groups still exhibited reduced CHIKV RNA levels and no detectable infectious virus in plasma, indicating that partial protective immunity may occur even at neutralizing antibody titers lower than 100. Further, human antibody Fc properties do not fully transfer to macaques due to differences in Fc gamma receptors, IgG subclasses, and glycosylation patterns^48,49^, suggesting that the protective antibody titer may need to be higher in cynomolgus macaques than in humans. The challenge dose of 10^7^ PFU we used also exceeds doses transmitted by mosquito vectors^33,50^, which suggests that partial protection observed in this high-dose animal model recapitulating severe CHIK disease that shows rapid and high peak viremia may translate to more pronounced clinical benefits in humans. Collectively, data from Studies B and C support the conclusion that neutralizing antibodies are sufficient to mediate vaccine-induced protection. Whether cell-mediated immune responses and non-neutralizing antibody also contribute to vaccine protection warrants further studies. For example, although T cell responses are known to modulate CHIK disease^51,52^, the studies presented here did not directly evaluate vaccine-induced T cell immunity.

Our study has several limitations, some of which have been circumvented by clinical trials conducted in the interim between our study and the present time. Although we observed a trend indicating greater protection with higher doses of the CHIKV VLP vaccine and inclusion of aluminum hydroxide adjuvant, the small animal group sizes meant that the differences between vaccine groups were not statistically significant. Clinical trials have addressed this question; they show that inclusion of the aluminum hydroxide adjuvant increased CHIKV neutralizing antibody GMT after the first but not second injection in 2-dose treatment groups^28^. The window between vaccinations (0, 28 days) and challenge (56 days) was a relatively short duration. This limited our ability to assess any differences in durability of efficacy between the groups at extended periods post-vaccination. All animals in our studies were healthy adults with no known prior exposure to alphaviruses. Clinical trials have shown that the CHIKV VLP is well tolerated and immunogenic in CHIKV seropositive people^28^ and in those who previously received an investigational vaccine for another alphavirus, Venezuelan equine encephalitis virus^53^. Animals in these studies received non-steroidal anti-inflammatory drugs, including ketoprofen, for animal welfare reasons, which may have attenuated clinical manifestations of arthritic disease and thus limited the sensitivity of clinical readouts used to assess vaccine-mediated protection. Lastly, we administered 2 doses of the CHIKV VLP vaccine 28 days apart, while more recent Phase III clinical trials (NCT05072080) in adolescents and adults have focused on a single dose^30,54^. This difference in dosing schedules makes it difficult to directly compare our results with those from Phase III human trials. However, we observed protection even with a very low dose of 1.25 μg adjuvanted vaccine, compared to the 40 μg adjuvanted vaccine used in humans. Despite these limitations, the animal studies provided important proof-of-concept that can guide future studies.

Overall, these studies demonstrate that the CHIKV VLP vaccine is immunogenic and provides protective efficacy against CHIKV in cynomolgus macaques. A dose as low as 1.25 μg of CHIKV VLP with aluminum hydroxide adjuvant and passively transferred IgG from CHIKV VLP-vaccinated humans significantly reduced viremia, disease, and joint pathology. The results also highlight the key role of neutralizing antibodies derived from vaccinated humans in mediating protection, even at lower antibody levels than the established protective PRNT_80_ threshold of ≥100. These findings demonstrate immunogenicity and protective efficacy of the CHIKV VLP and support use of this vaccine to protect humans against CHIK disease in endemic areas to prevent or rapidly curtail outbreaks and to protect travelers to endemic countries.

## Supplementary information

Supplementary Information

Chikv Nhp Vlp Raw Data

## Data Availability

The data are available in the Supplemental File ‘CHIKV NHP VLP raw data’.
