# Supplementary material for: A chikungunya virus-like particle vaccine reduces chikungunya disease in cynomolgus macaques and protection is mediated by antibody transferred from vaccinated humans
Source: NPJ Vaccines. 2026 Mar 18;11:97. doi: 10.1038/s41541-026-01413-z (PMC13149838; doi:10.1038/s41541-026-01413-z)
Supplement: Supplementary file 1 — Supplementary Information [file 41541_2026_1413_MOESM1_ESM.pdf]

A chikungunya virus-like particle vaccine reduces chikungunya disease in cynomolgus macaques and protection is mediated by antibody transferred from vaccinated humans

SUPPLEMENTAL INFORMATION

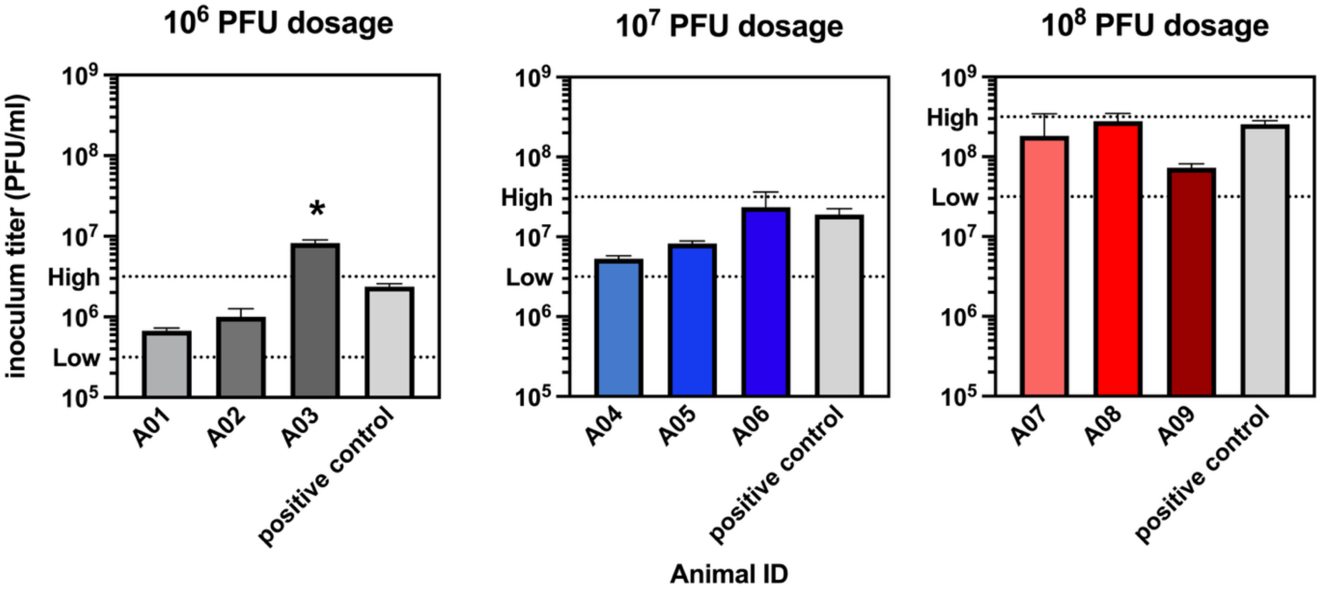

Supplemental Figure 1: Back-titration of CHIKV LR-OPY1 inocula for Study A. On days of virus challenge, the remainder of virus stock in the vial administered to animals was refrozen at -80°C and then processed as a batch by thawing and titrating on Vero cells. Animals were inoculated with 0.42, 0.52 or 0.39 ml of the 10^6, 10^7 or 10^8 PFU vials, to reach the desired inoculum dose, which was verified for each animal (A01-A09). Dotted lines indicate the 10-fold range around the titer of the positive control, which was thawed and re-frozen to reproduce the same treatment as the virus used in inocula. The asterisk above A03 shows the titer was above the 10-fold range around the target dose. PFU is plaque forming units.

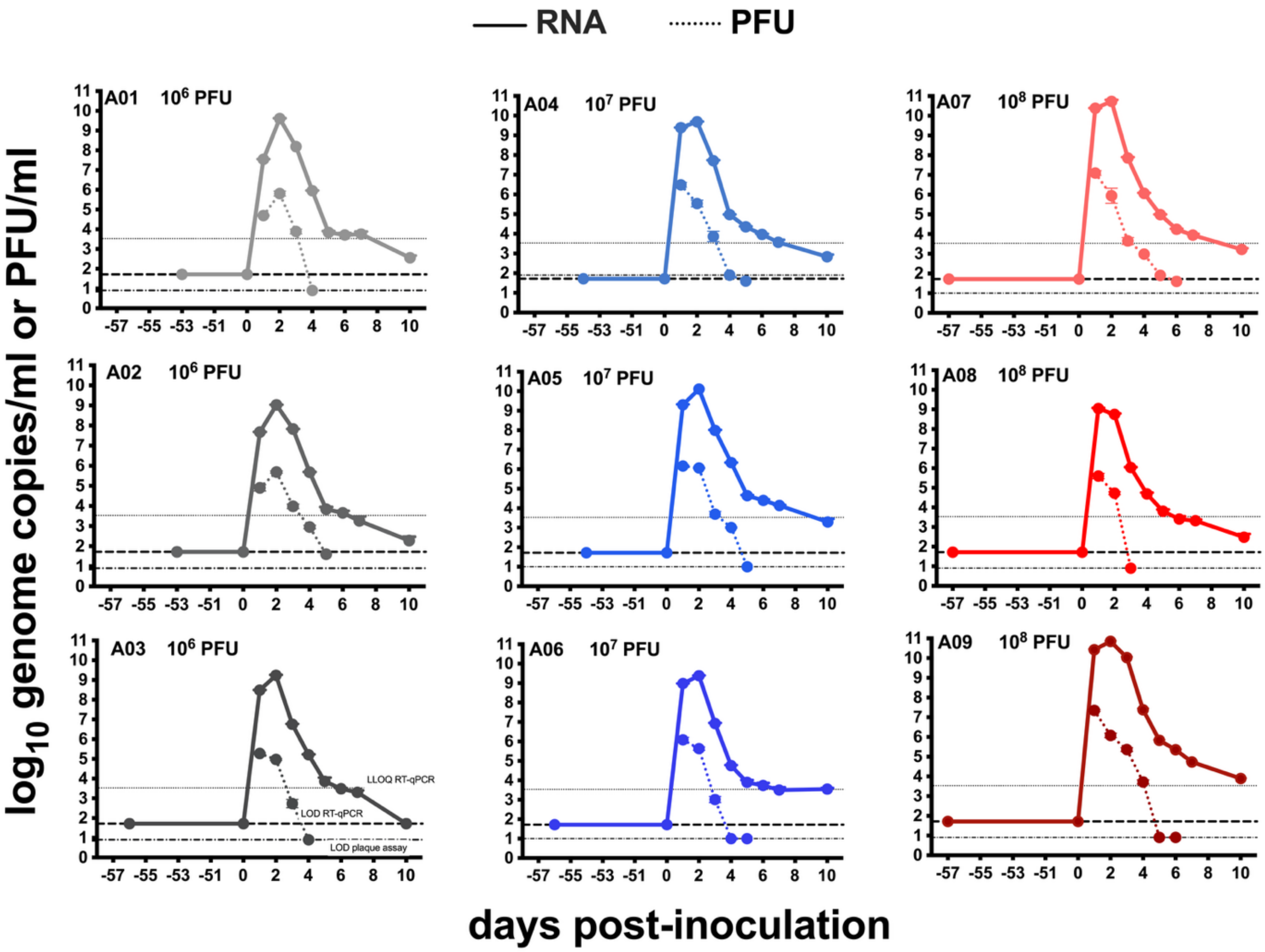

Supplemental Figure 2: CHIKV plasma viremia kinetics in cynomolgus macaques inoculated with 10^6, 10^7, or 10^8 PFU on a per animal level. The solid lines show CHIKV RNA levels and the dotted lines show CHIKV PFU levels. Each dot represents the mean of triplicate technical RT-qPCR replicates and duplicate titrations. The LOD and LOQ are indicated by horizontal lines. Samples that were undetectable were given a value of the LOD for graphing purposes. PFU is plaque forming units.

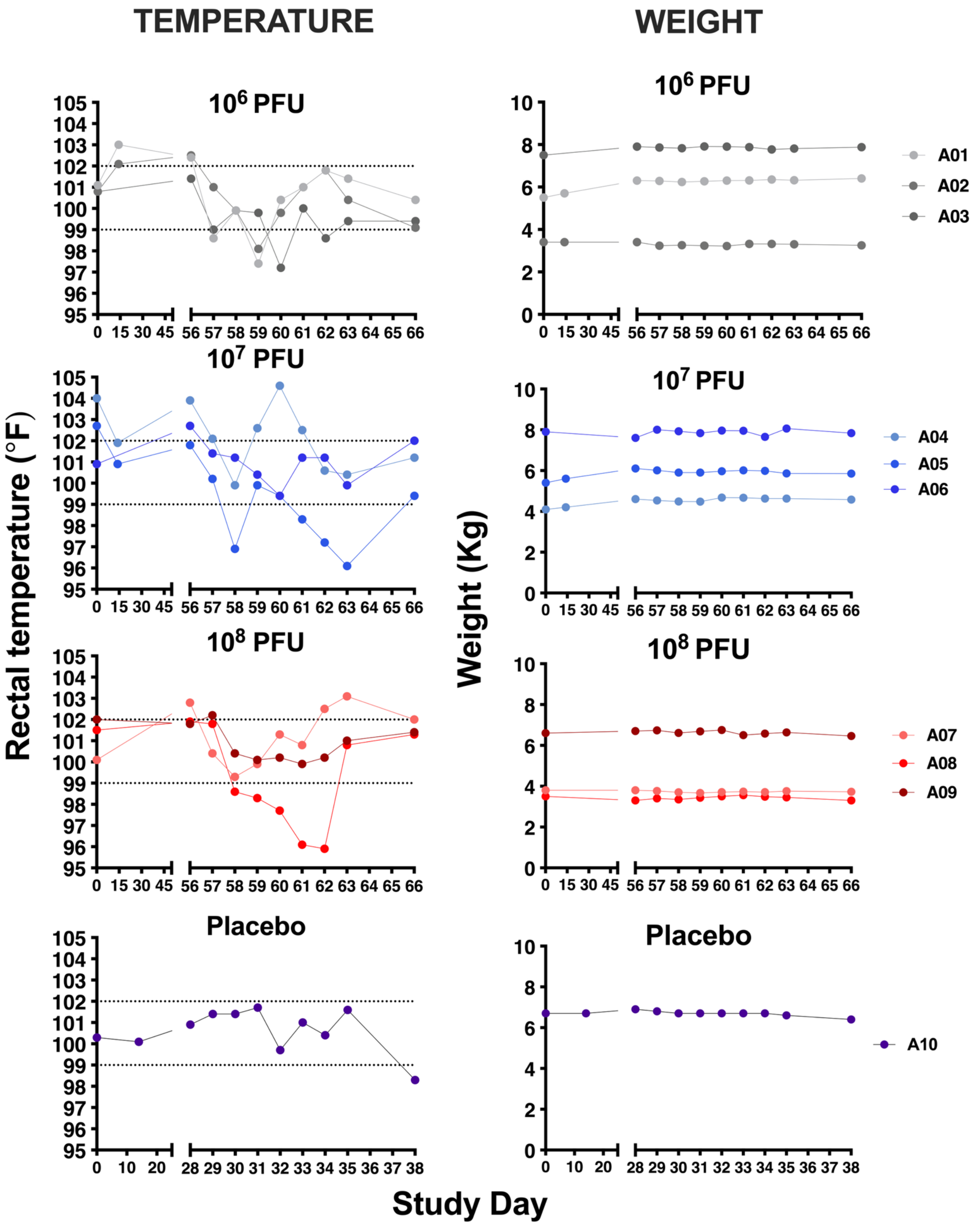

TEMPERATURE
WEIGHT
10^6 PFU
10^7 PFU
10^8 PFU
Placebo
Rectal temperature (°F)
Weight (Kg)
Study Day
A01
A02
A03
A04
A05
A06
A07
A08
A09
A10

Supplemental Figure 3: Time course of rectal temperatures and body weights in CHIKV- or placebo-inoculated cynomolgus macaques. The 3 CHIKV-infected animal groups were inoculated on study day 56 and monitored until day 66. The placebo-treated animal was mock-inoculated on study day 28 and monitored until day 38. Dotted lines indicate the normal temperature range (99-102 °F).

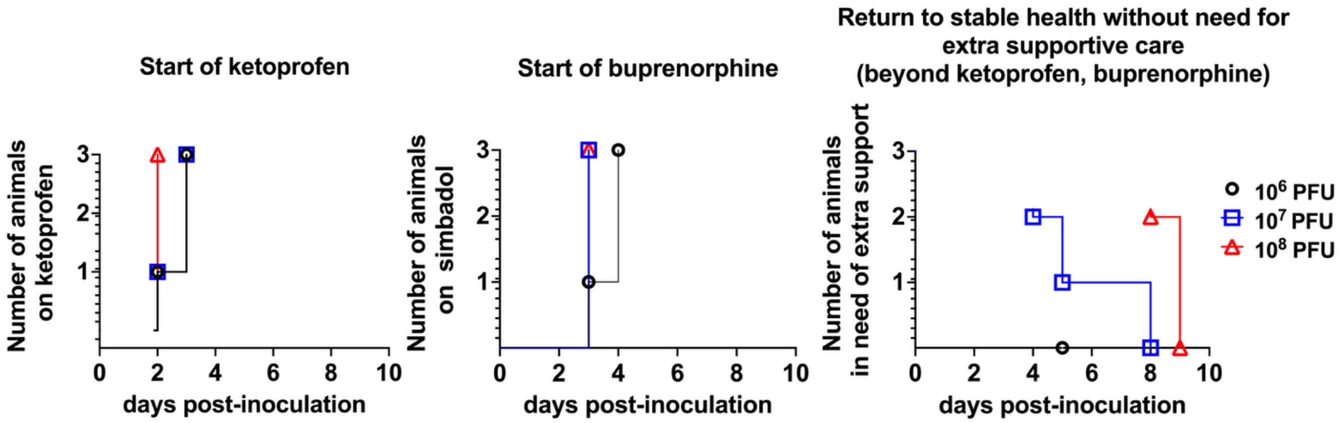

Supplemental Figure 4: Initiation and duration of supportive care in CHIKV-inoculated cynomolgus macaques. Days of treatment initiation of the analgesics ketoprofen and long-acting buprenorphine for pain management and time when animals returned to normal clinical status with normal appetite and no longer required other supportive care beyond ketoprofen or buprenorphine including subcutaneous fluids or orogastric intubation for nutritional supplementation. Differences across groups were not statistically significant ($p>0.05$, Logrank test).

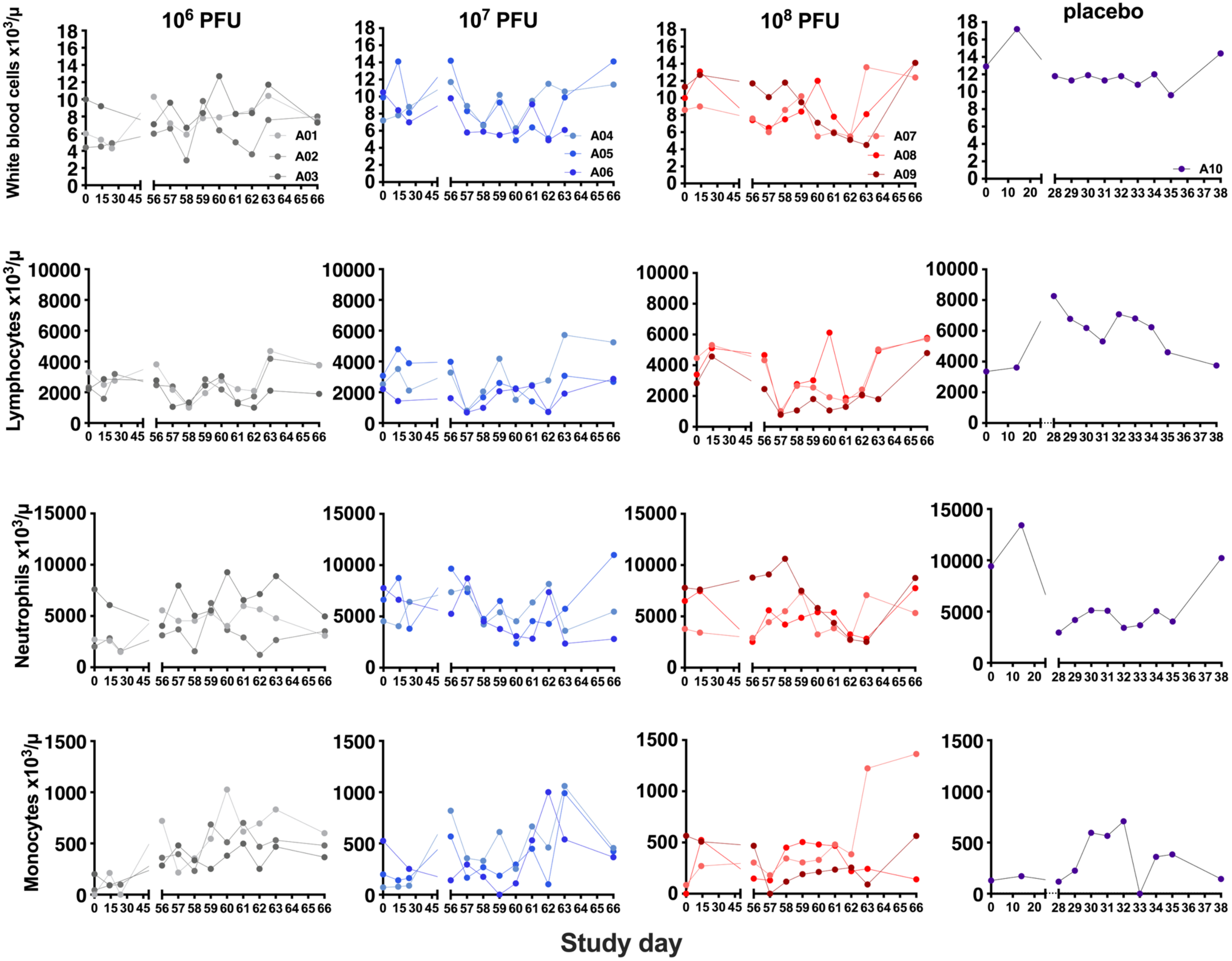

10^6 PFU
10^7 PFU
10^8 PFU
placebo
White blood cells x10^3/µ
Lymphocytes x10^3/µ
Neutrophils x10^3/µ
Monocytes x10^3/µ
A01
A02
A03
A04
A05
A06
A07
A08
A09
A10
Study day

Supplemental Figure 5: **Time course of white blood cells, lymphocytes, neutrophils, and monocytes in CHIKV-inoculated or placebo-treated cynomolgus macaques**.

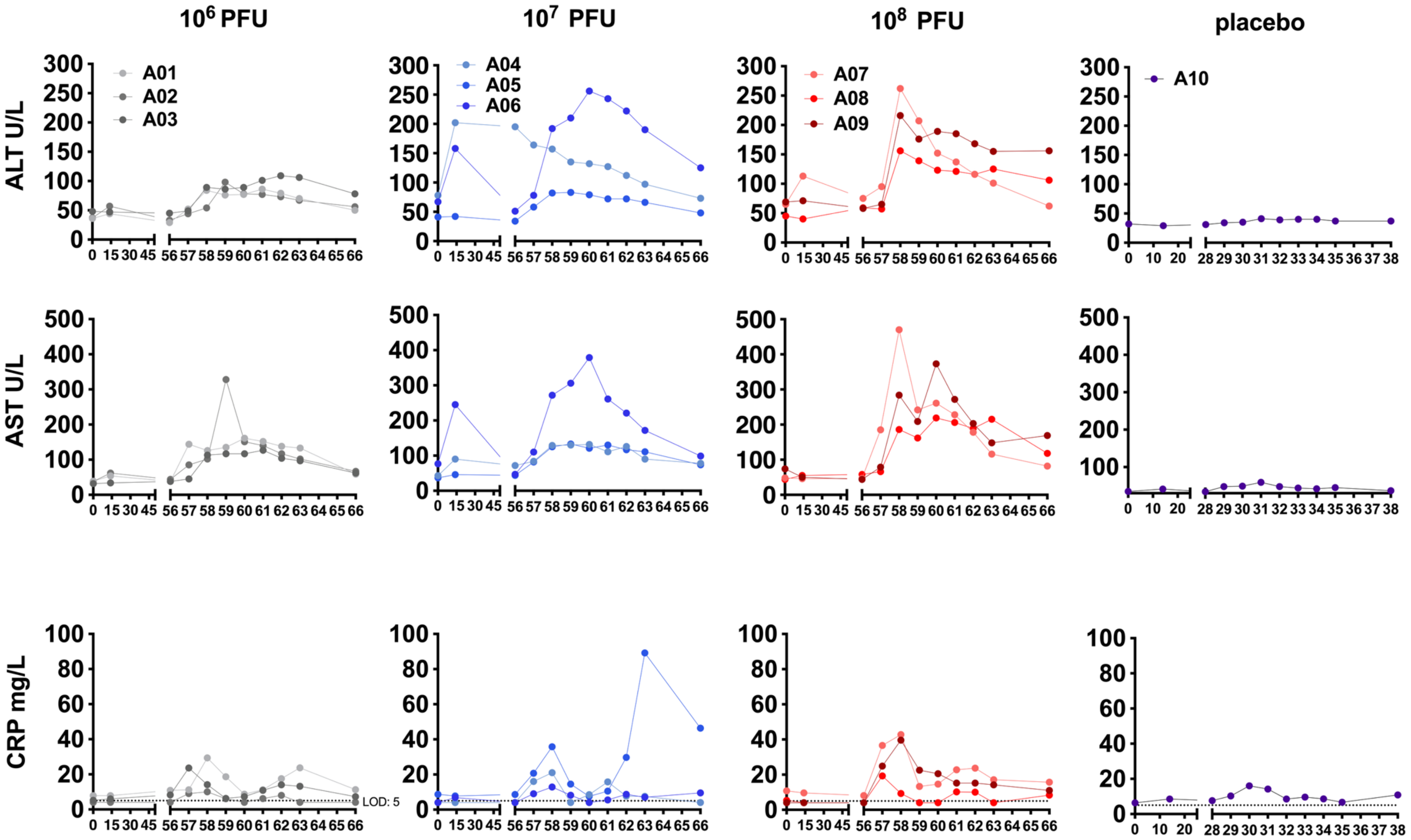

10^6 PFU
10^7 PFU
10^8 PFU
placebo
ALT U/L
AST U/L
CRP mg/L
A01
A02
A03
A04
A05
A06
A07
A08
A09
A10
LOD: 5

Supplemental Figure 6: **Time course of alanine transaminase (ALT), aspartate transaminase (AST), and C-reactive protein (CRP) in serum of CHIKV-inoculated or placebo-treated cynomolgus macaques.**

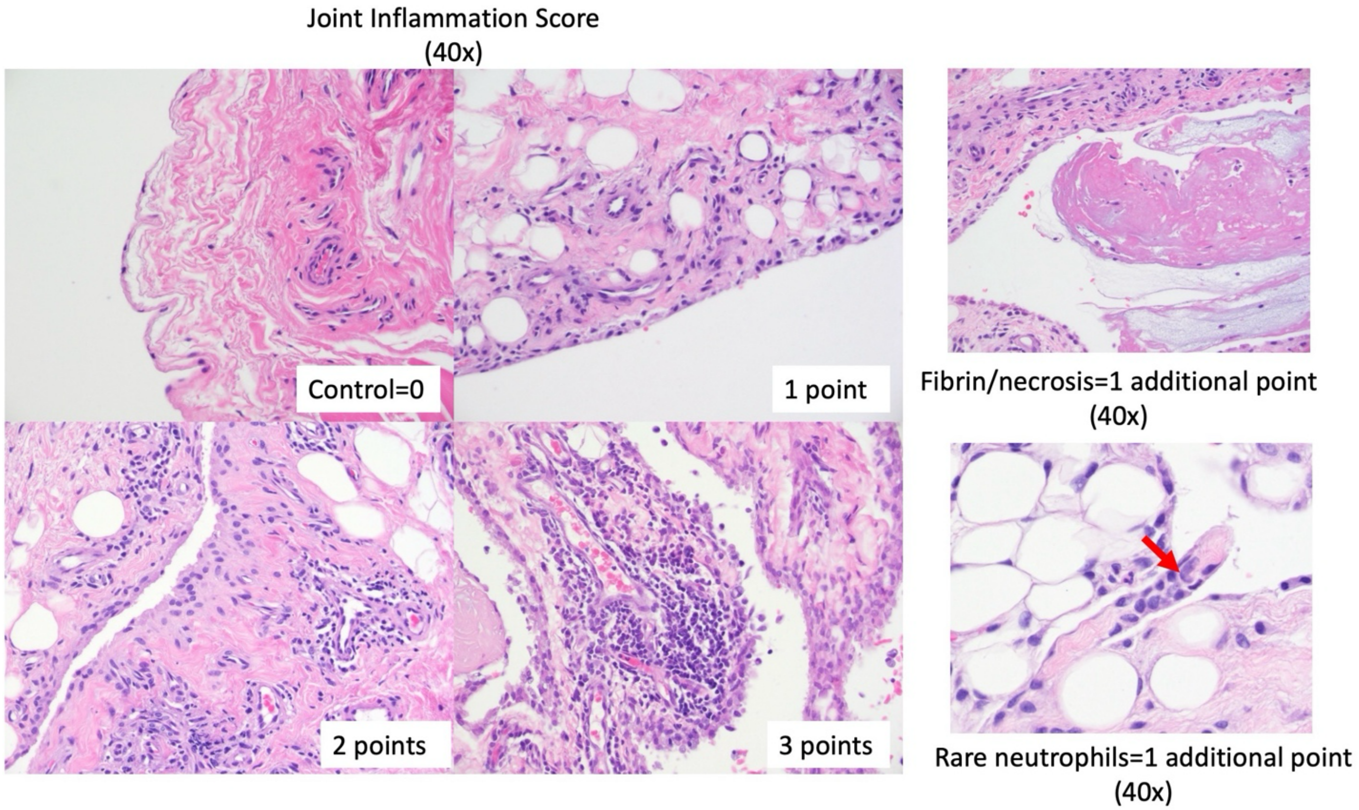

Supplemental Figure 7: **Representative histopathology images encompassing the range of severity of joint inflammation across the 4 point scoring scale**. Tissues are stained with hematoxylin and eosin.

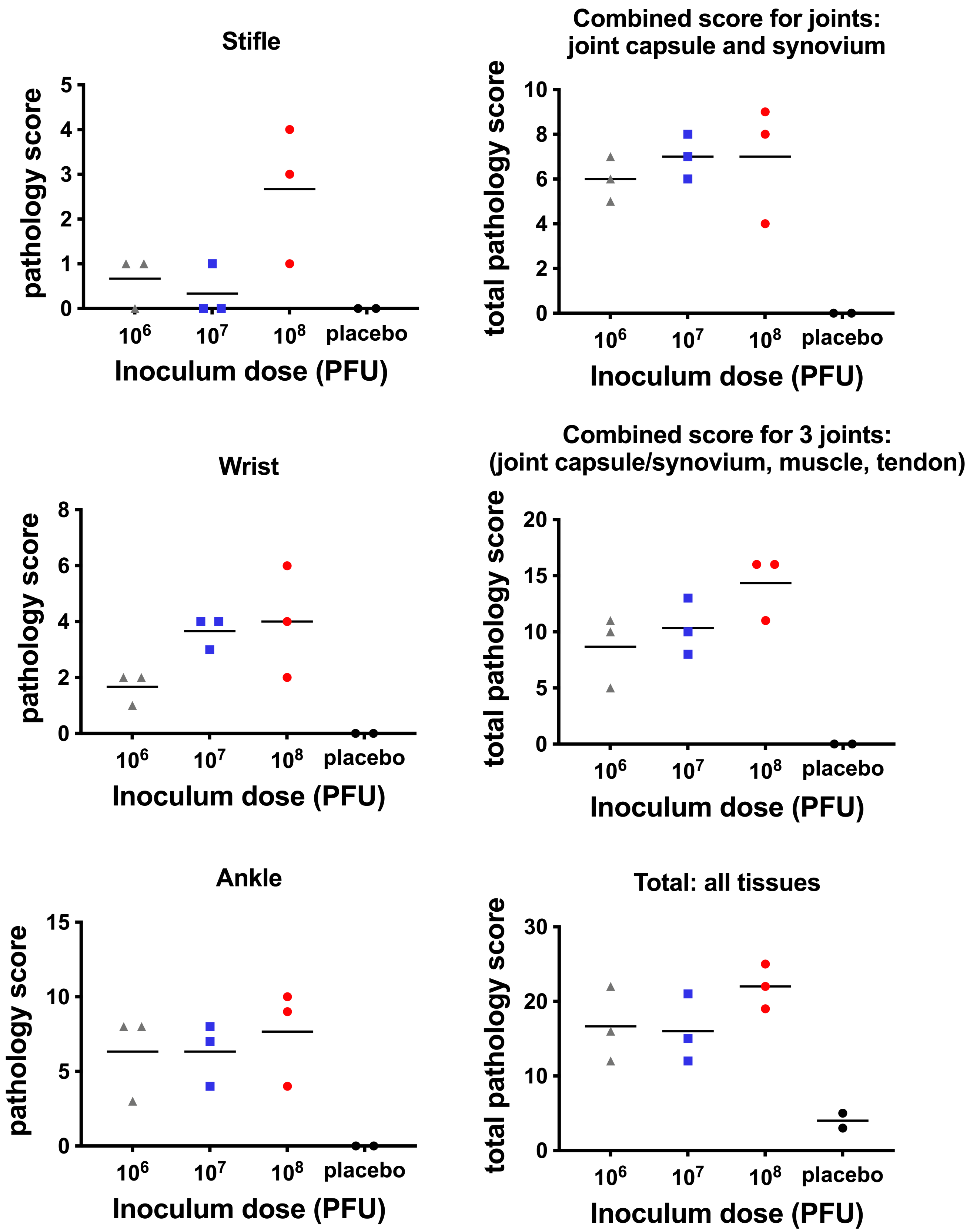
Stifle
pathology score
5
4
3
2
1
0
10^6
10^7
10^8
placebo
Inoculum dose (PFU)
Combined score for joints:
joint capsule and synovium
total pathology score
10
8
6
4
2
0
10^6
10^7
10^8
placebo
Inoculum dose (PFU)
Wrist
pathology score
8
6
4
2
0
10^6
10^7
10^8
placebo
Inoculum dose (PFU)
Combined score for 3 joints:
(joint capsule/synovium, muscle, tendon)
total pathology score
20
15
10
5
0
10^6
10^7
10^8
placebo
Inoculum dose (PFU)
Ankle
pathology score
15
10
5
0
10^6
10^7
10^8
placebo
Inoculum dose (PFU)
Total: all tissues
total pathology score
30
20
10
0
10^6
10^7
10^8
placebo
Inoculum dose (PFU)

Supplemental Figure 8: Histopathology scores in joints of CHIKV-inoculated or placebo-treated cynomolgus macaques. The stifle, ankle, and wrist were scored for combined histological findings in joint capsule/synovium, and adjacent tendon and muscle using a scoring metric (**Table 2**). The sum of all three joint scores for each animal is the combined score for three joints. The combined scores of joint capsule and synovium only exclude scores for tendon and muscle. The total pathology score of all tissues includes the lymphoid tissues and major organs. Horizontal lines indicate the mean. Analyses used Kruskal-Wallis tests.

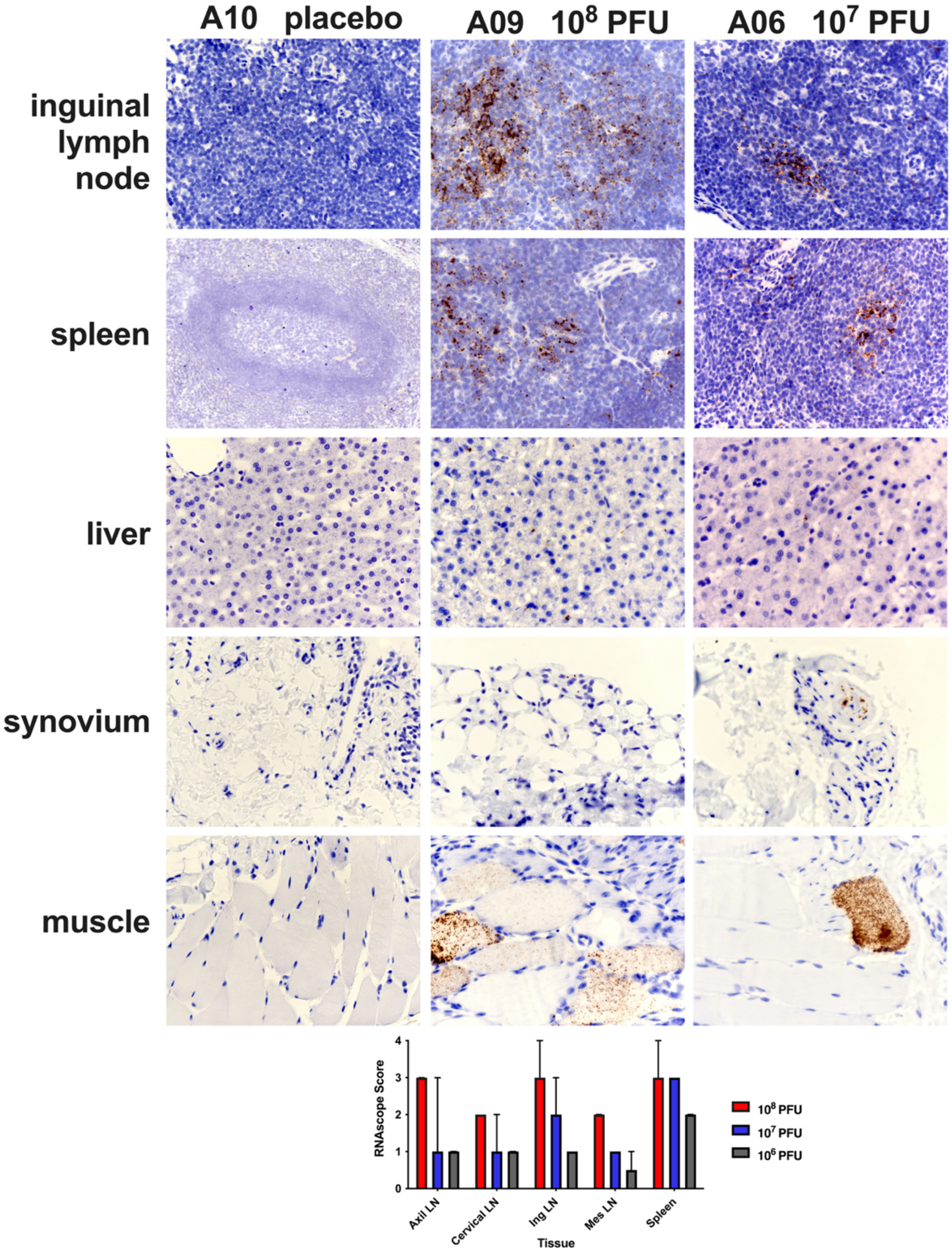

A10 placebo
A09 10^8 PFU
A06 10^7 PFU
inguinal lymph node
spleen
liver
synovium
muscle
RNAscope Score
Axil LN
Cervical LN
Ing LN
Mes LN
Spleen
Tissue
10^8 PFU
10^7 PFU
10^6 PFU

Supplemental Figure 9: CHIKV RNA in tissues of CHIKV- or placebo-inoculated cynomolgus macaques detected using *in situ* hybridization (ISH). Brown color indicates CHIKV RNA and cell nuclei are blue. RNAscope ISH scoring criteria for lymphoid tissues are presented in Table 2. The graph shows mean values. Kruskal-Wallis tests. Axil is axillary, Ing is inguinal, Mes is mesenteric. LN is lymph node. PFU is plaque forming units.

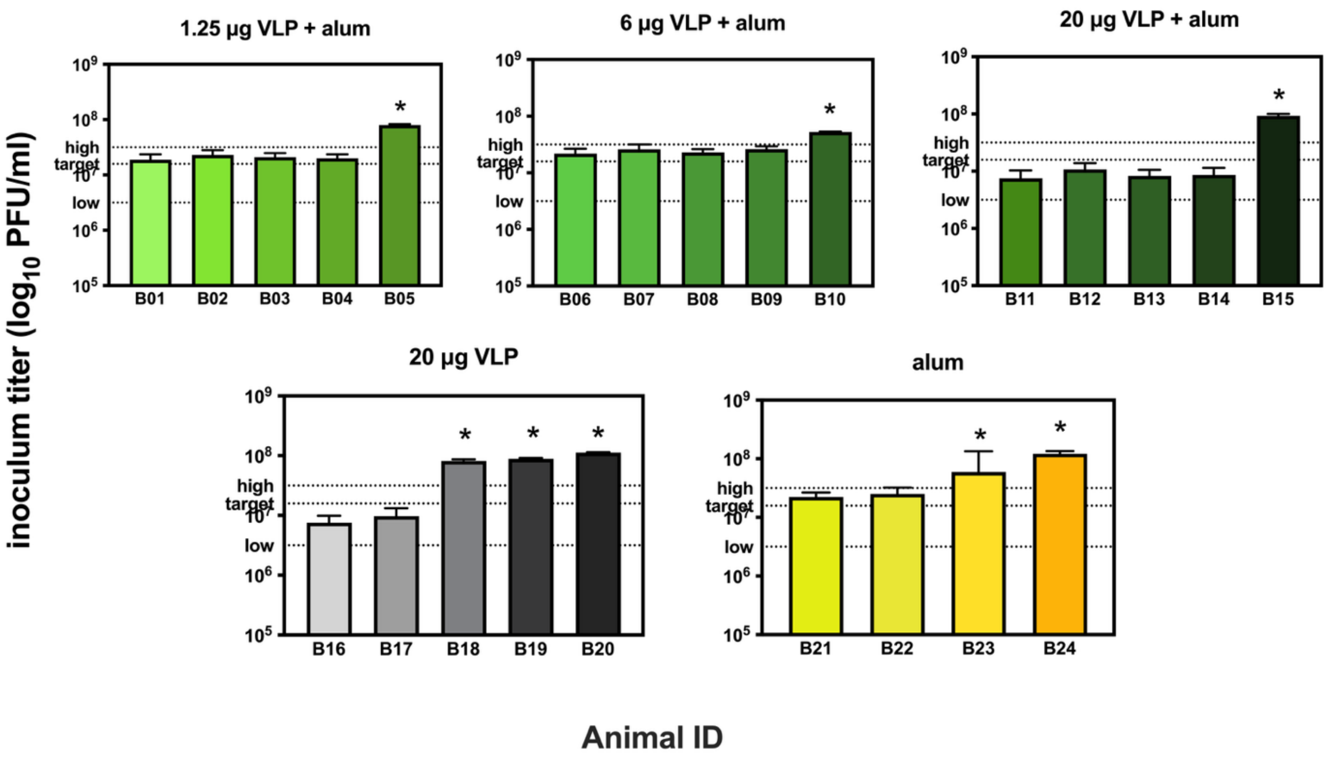

Supplemental Figure 10: Back-titration of CHIKV LR-OPY1 inocula for vaccine and challenge in Study B. The measured titer of the administered stock was 7.2 $\log_{10}$ PFU/ml and is shown as the 'target' line. Dotted low and high lines indicate the 10-fold range around the 10^7 target. The asterisks show values above the 10-fold range. PFU is plaque forming units.

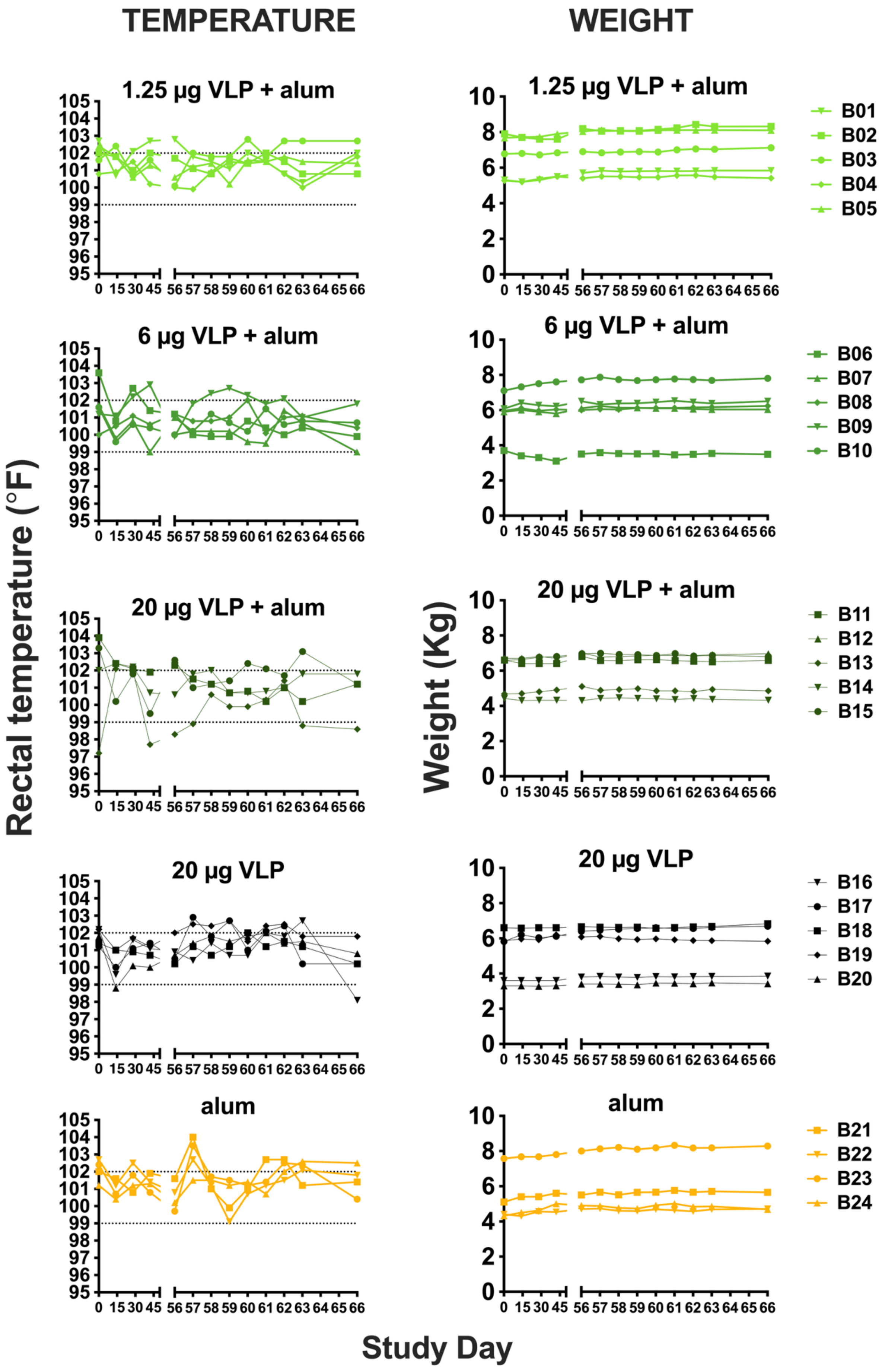
TEMPERATURE
WEIGHT
1.25 µg VLP + alum
6 µg VLP + alum
20 µg VLP + alum
20 µg VLP
alum
Rectal temperature (°F)
Weight (Kg)
Study Day
B01
B02
B03
B04
B05
B06
B07
B08
B09
B10
B11
B12
B13
B14
B15
B16
B17
B18
B19
B20
B21
B22
B23
B24

Supplemental Figure 11: Time course of rectal temperatures and body weights in CHIKV VLP + alum vaccinated or alum-only treated CHIKV-challenged cynomolgus macaques. Dotted lines indicate the normal temperature range (99-102 °F).

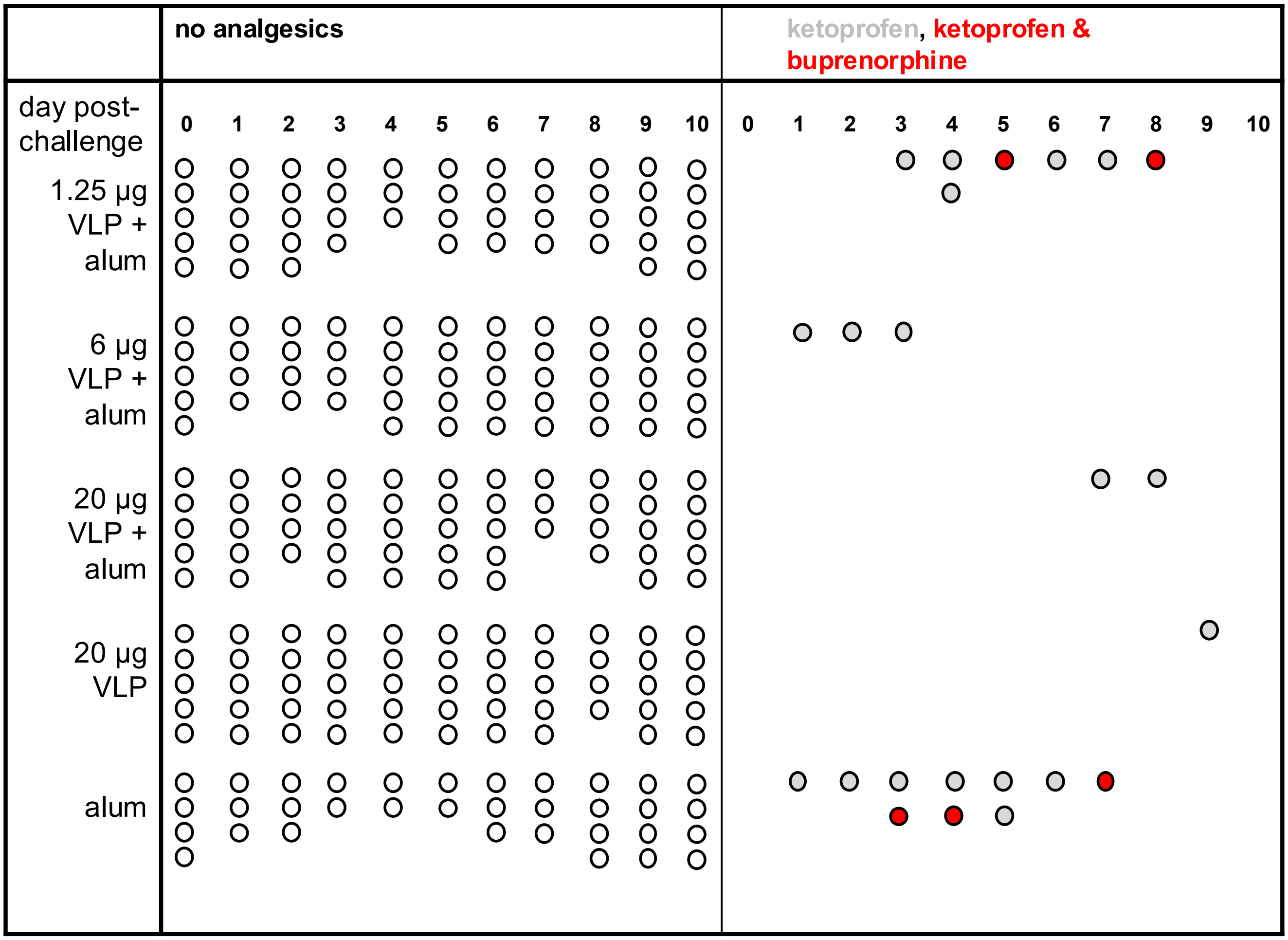

Supplemental Figure 12: Treatment with analgesics after vaccination and CHIKV challenge in cynomolgus macaques. Each animal is represented by a circle on each day post-challenge. The left side shows animals that did not receive any analgesic treatment. The right side shows animals that received ketoprofen (gray) or both ketoprofen and long-acting buprenorphine (red).

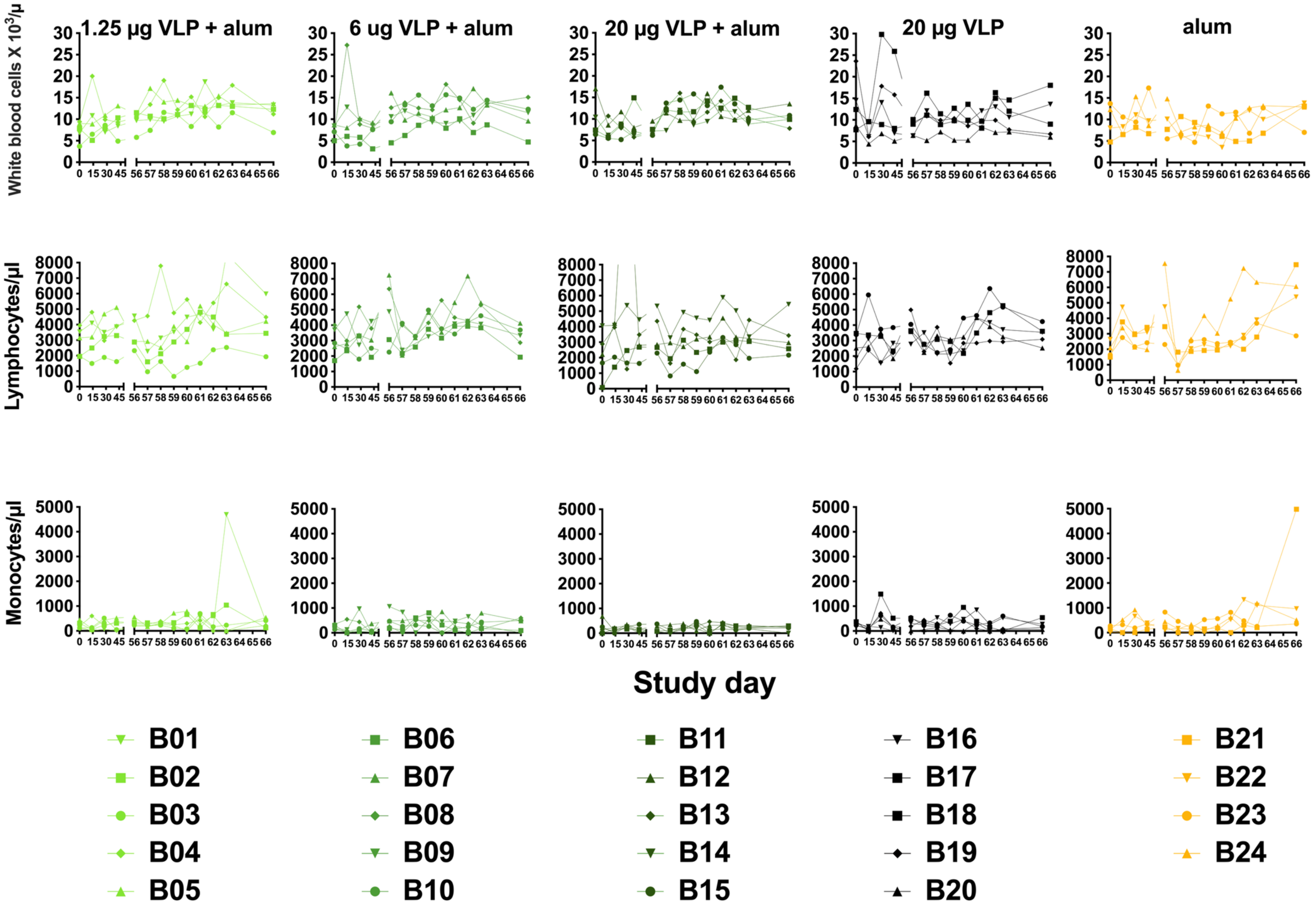
1.25 µg VLP + alum
6 ug VLP + alum
20 µg VLP + alum
20 µg VLP
alum
White blood cells X 10^3/µ
Lymphocytes/µl
Monocytes/µl
0 15 30 45 56 57 58 59 60 61 62 63 64 65 66
Study day
B01
B02
B03
B04
B05
B06
B07
B08
B09
B10
B11
B12
B13
B14
B15
B16
B17
B18
B19
B20
B21
B22
B23
B24

Supplemental Figure 13: **Time course of white blood cells, lymphocytes, and monocyte counts in vaccinated and CHIKV challenged cynomolgus macaques**.

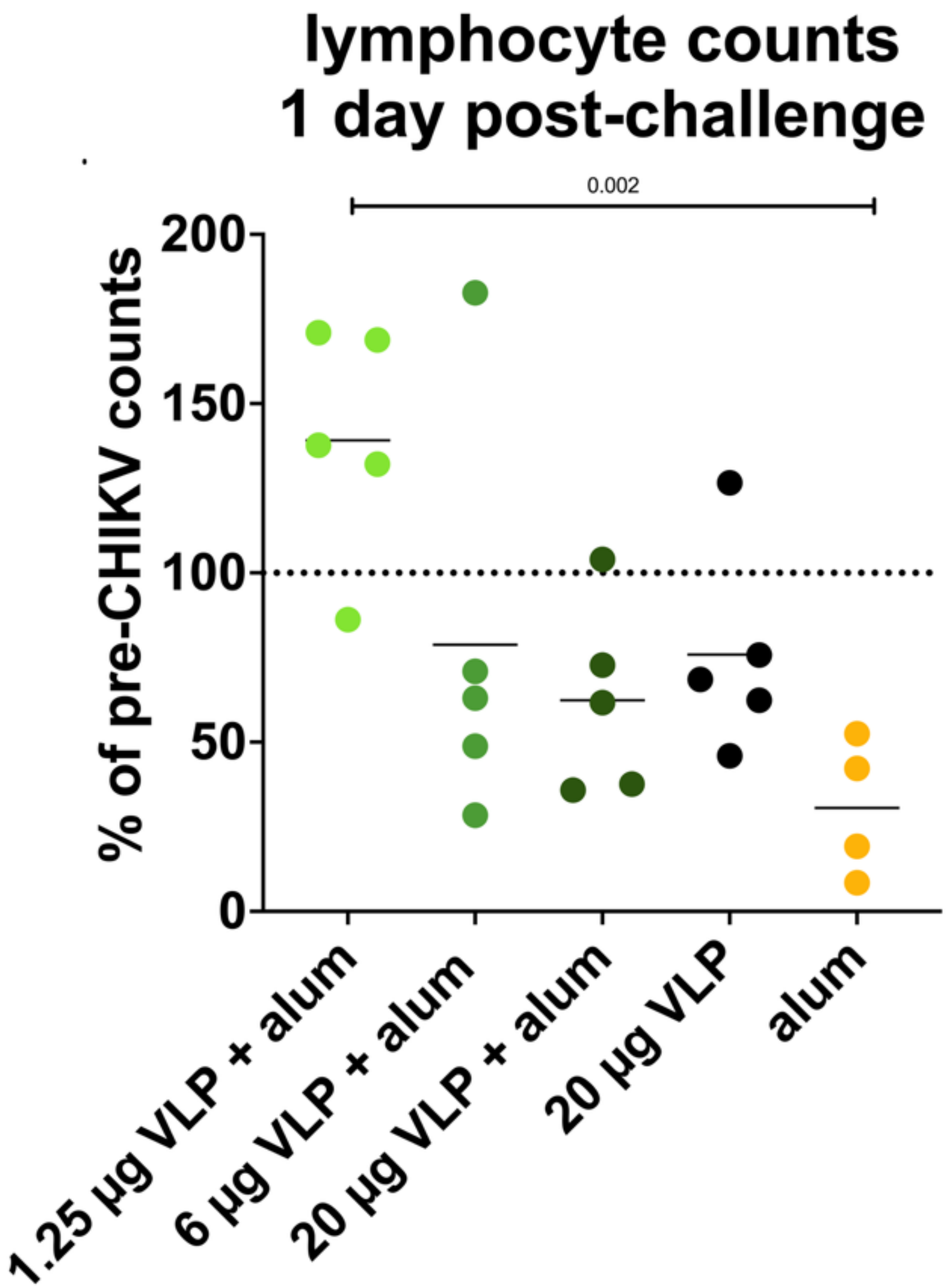

Supplemental Figure 14: **Lymphocyte counts on study day 57, 1 day after virus inoculation in vaccinated or alum treated and CHIKV challenged cynomolgus macaques.** Counts are expressed as percentage of the lymphocyte numbers on study day 56, just prior to CHIKV inoculation. Lines represent means. Groups were significantly different (p=0.006, ANOVA) and this was driven by a differences between alum versus 1.25 µg VLP + alum groups (p=0.002, Holmes-Sidak's multiple comparison's test).

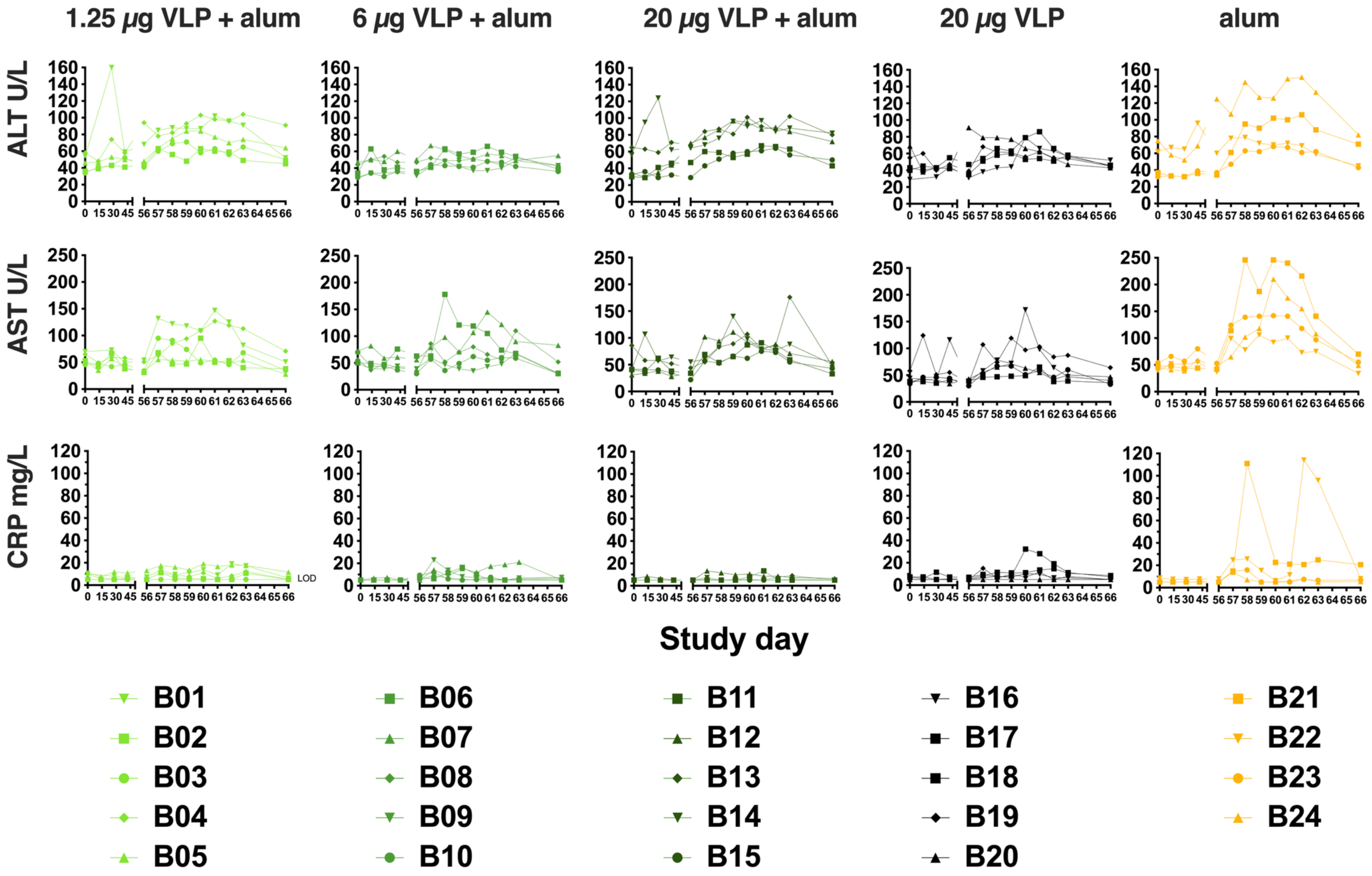
1.25 μg VLP + alum
6 μg VLP + alum
20 μg VLP + alum
20 μg VLP
alum
ALT U/L
AST U/L
CRP mg/L
LOD
Study day
B01
B02
B03
B04
B05
B06
B07
B08
B09
B10
B11
B12
B13
B14
B15
B16
B17
B18
B19
B20
B21
B22
B23
B24

Supplemental Figure 15: Time course of alanine transaminase (ALT), aspartate transaminase (AST), and C-reactive protein (CRP) in serum of vaccinated or alum treated and CHIKV challenged cynomolgus macaques.

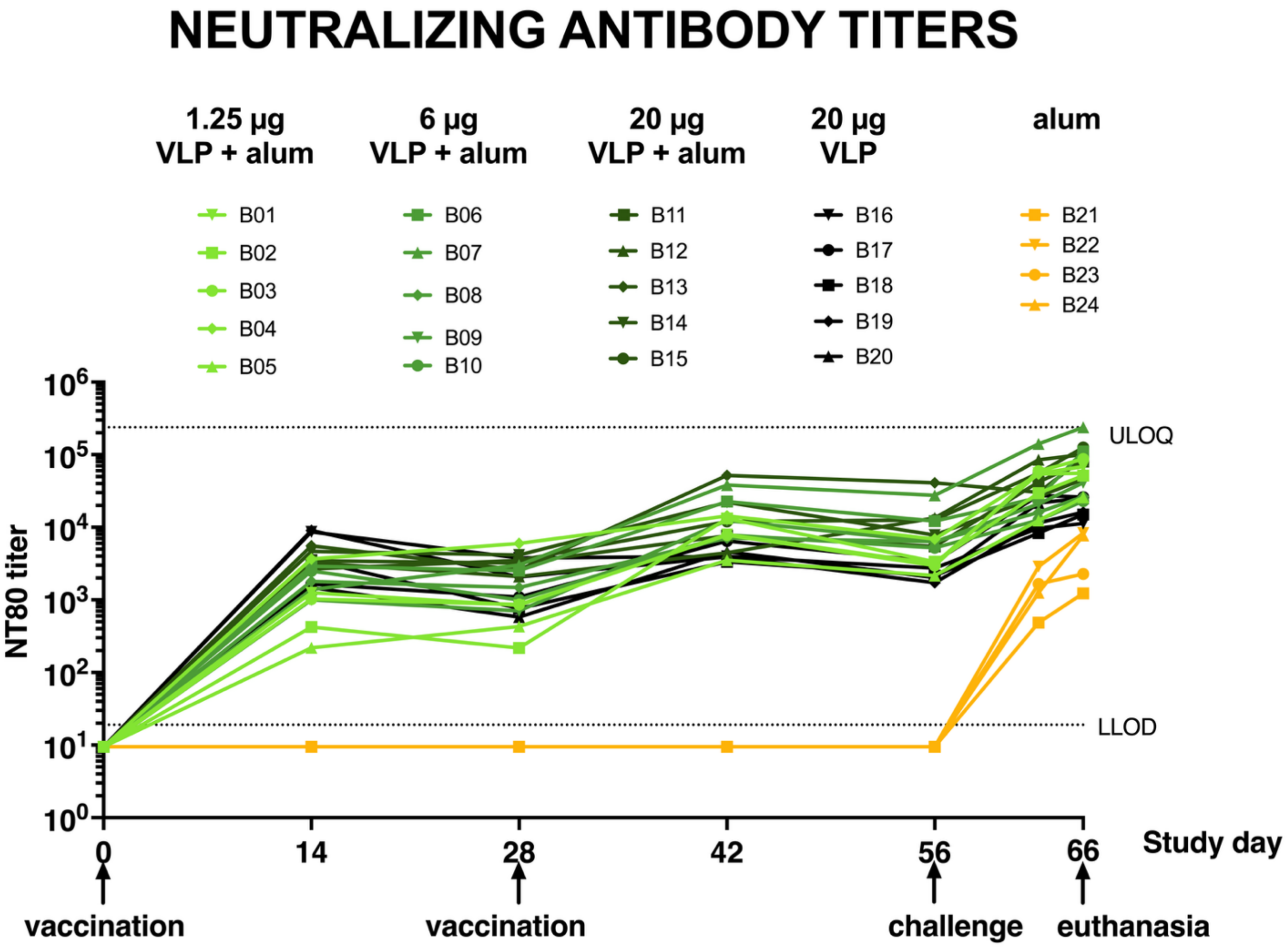

Supplemental Figure 16: Temporal kinetics of CHIKV neutralizing antibody titers after vaccination or alum treatment and CHIKV challenge in individual cynomolgus. Each line represents one animal. Neutralization titers for each sample were generated from duplicate NT_{80} assays and are reported as the average. ULOD is upper limit of quantification. LLOD is the lower limit of detection.

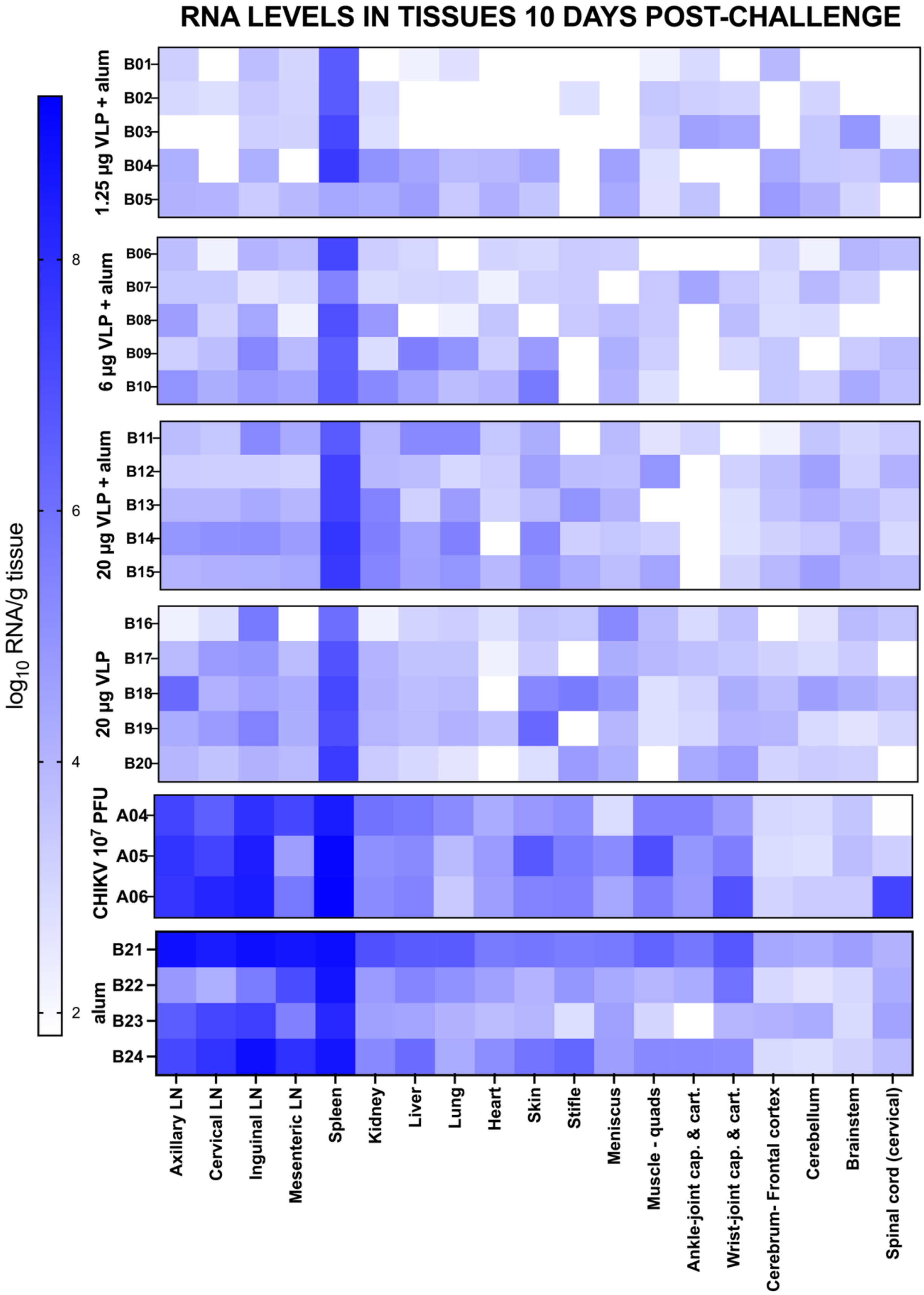

RNA LEVELS IN TISSUES 10 DAYS POST-CHALLENGE
log10 RNA/g tissue
2
4
6
8
1.25 µg VLP + alum
B01
B02
B03
B04
B05
6 µg VLP + alum
B06
B07
B08
B09
B10
20 µg VLP + alum
B11
B12
B13
B14
B15
20 µg VLP
B16
B17
B18
B19
B20
CHIKV 10^7 PFU
A04
A05
A06
alum
B21
B22
B23
B24
Axillary LN
Cervical LN
Inguinal LN
Mesenteric LN
Spleen
Kidney
Liver
Lung
Heart
Skin
Stifle
Meniscus
Muscle - quads
Ankle-joint cap. & cart.
Wrist-joint cap. & cart.
Cerebrum- Frontal cortex
Cerebellum
Brainstem
Spinal cord (cervical)

Supplemental Figure 17: CHIKV RNA levels in all sampled tissues after vaccination or alum treatment and CHIKV challenge in cynomolgus macaques. CHIKV RNA levels were measured in homogenized tissues sampled at necropsy, 10 days post-challenge. Darker colors show increasing CHIKV RNA levels. Each CHIKV RNA measurement was generated from triplicate RT-qPCR assays and is reported as the average. LN is lymph node. Quads is quadricep. Cap. Is capsule. Cart. Is cartilage.

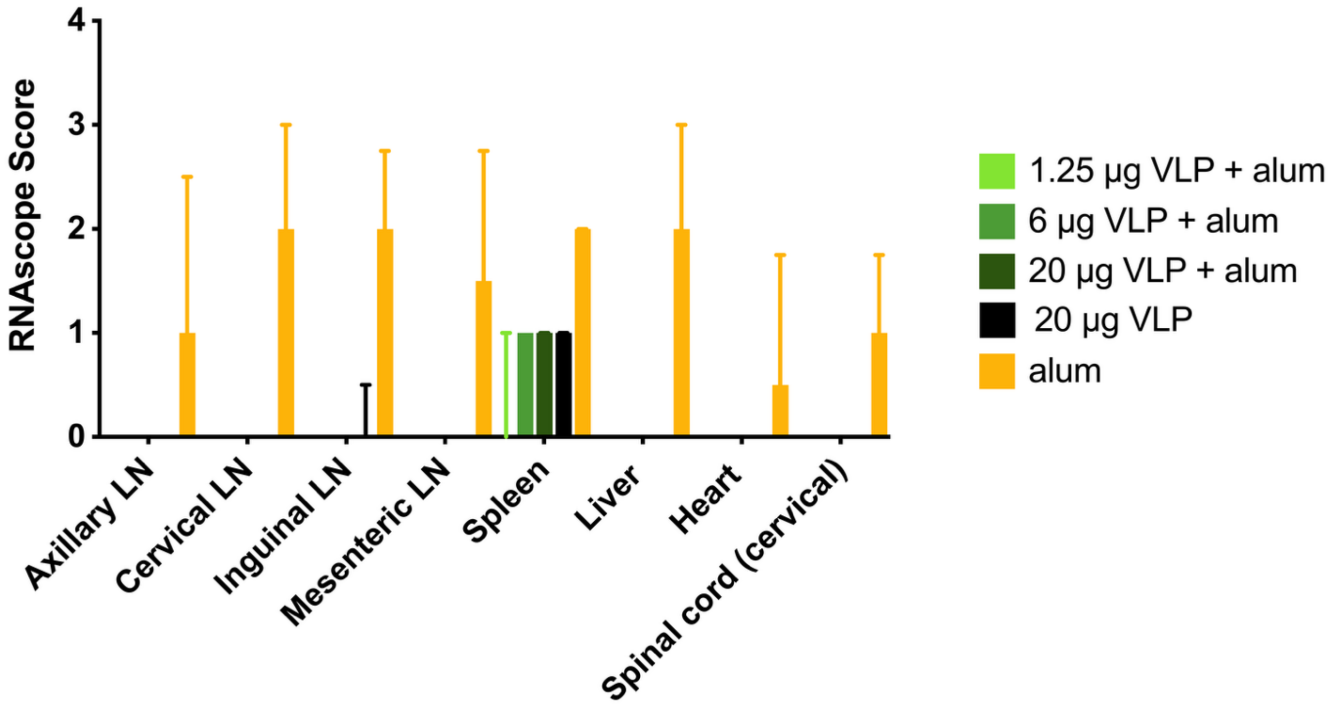

Supplemental Figure 18: CHIKV RNA in tissues of CHIKV VLP vaccinated or alum treated CHIKV-inoculated cynomolgus macaques detected using *in situ* hybridization (ISH). RNAscope ISH scoring criteria for lymphoid tissues are presented in Table 3. The graph shows mean values with error bars showing ranges. Kruskal-Wallis tests were used.

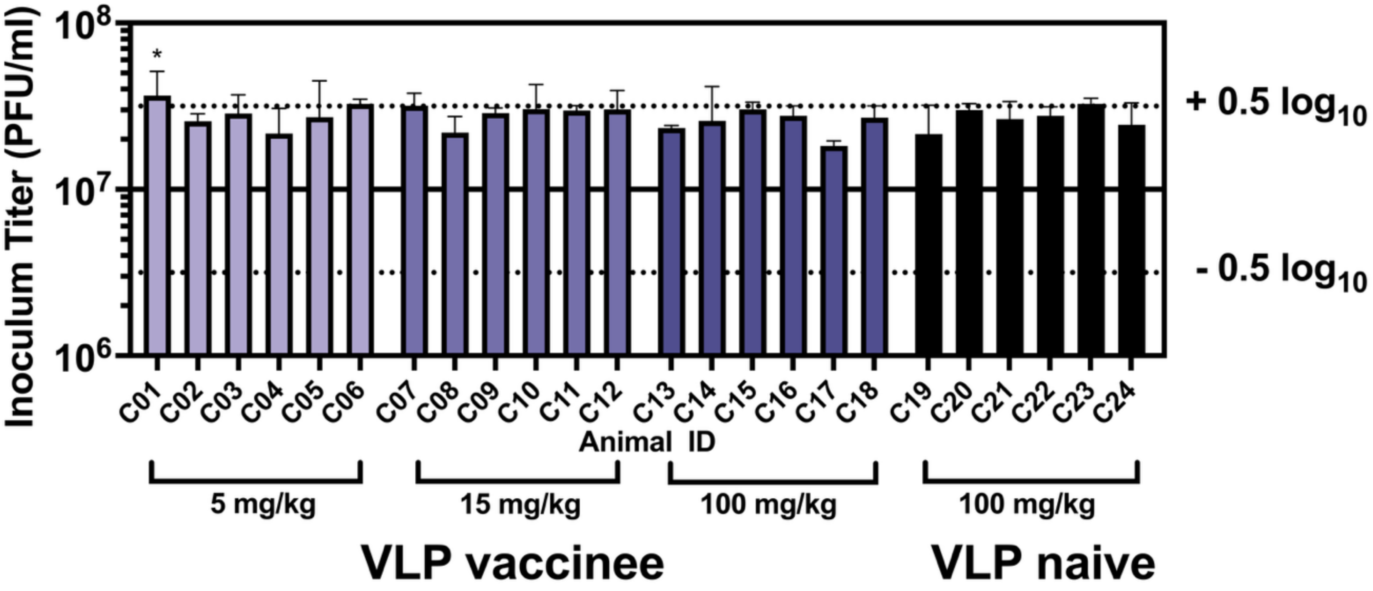

Supplemental Figure 19: Back-titration of CHIKV LR-OPY1 inocula for passive IgG transfer (Study C). Dotted lines indicate the 0.5 log_{10} range around the goal dose of 10^7 PFU. The asterisk above animal C01 shows the one inoculum whose value was above the 0.5 log_{10} range. PFU is plaque forming units.

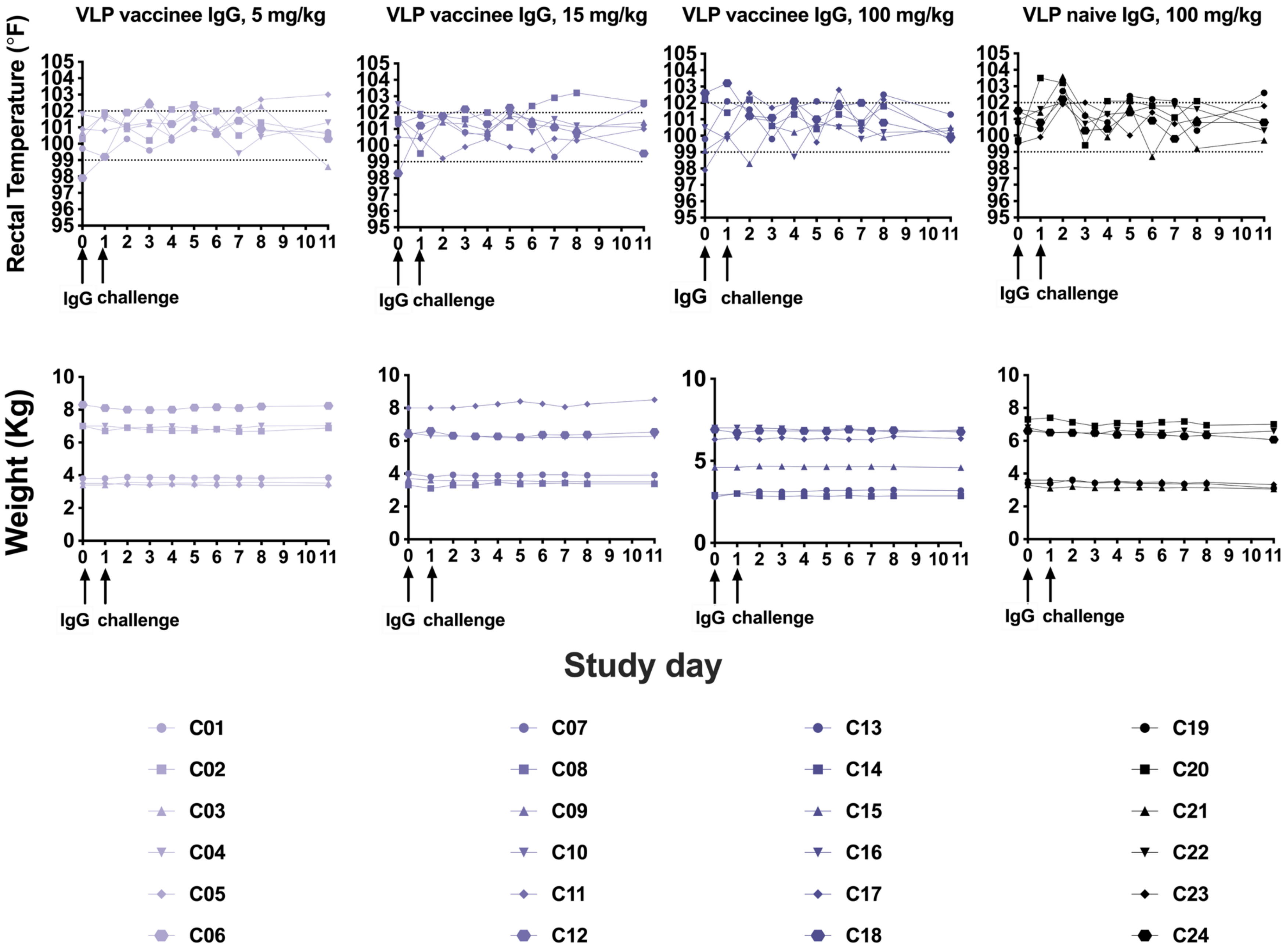

VLP vaccinee IgG, 5 mg/kg
VLP vaccinee IgG, 15 mg/kg
VLP vaccinee IgG, 100 mg/kg
VLP naive IgG, 100 mg/kg
Rectal Temperature (°F)
Weight (Kg)
IgG
challenge
Study day
C01
C02
C03
C04
C05
C06
C07
C08
C09
C10
C11
C12
C13
C14
C15
C16
C17
C18
C19
C20
C21
C22
C23
C24

Supplemental Figure 20: **Time course of rectal temperatures and body weights in cynomolgus macaques administered antibody from CHIKV VLP vaccinees or VLP naïve people**. Dotted lines indicate the normal temperature range (99-102 °F).

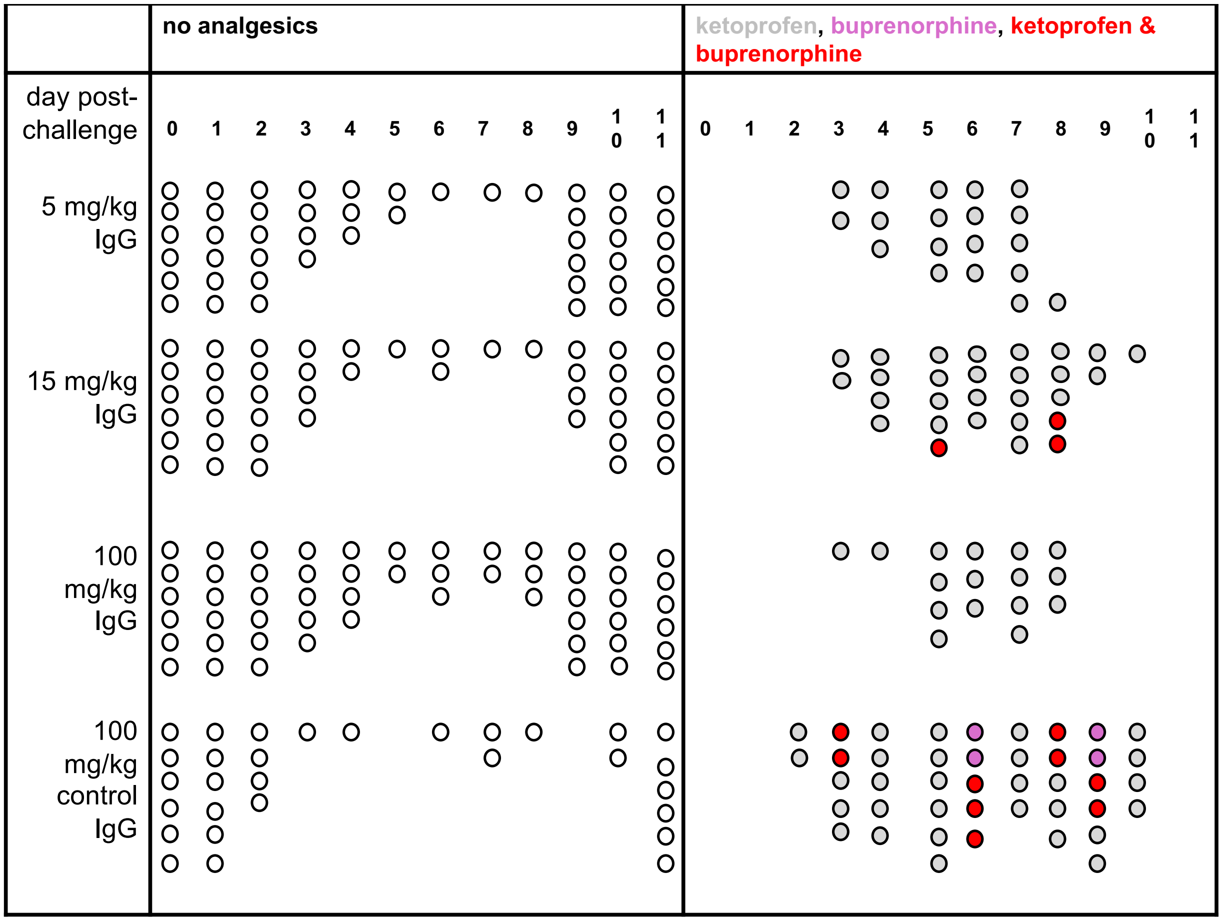

Supplemental Figure 21: Treatment with analgesics after IgG administration from CHIKV VLP vaccinated or naïve people and CHIKV challenge in cynomolgus macaques. Each animal is represented by a circle on each day post-challenge. The left side shows animals that did not receive any analgesic treatment. The right side shows animals that received ketoprofen (gray), long-acting buprenorphine (purple), or both ketoprofen and buprenorphine (red).

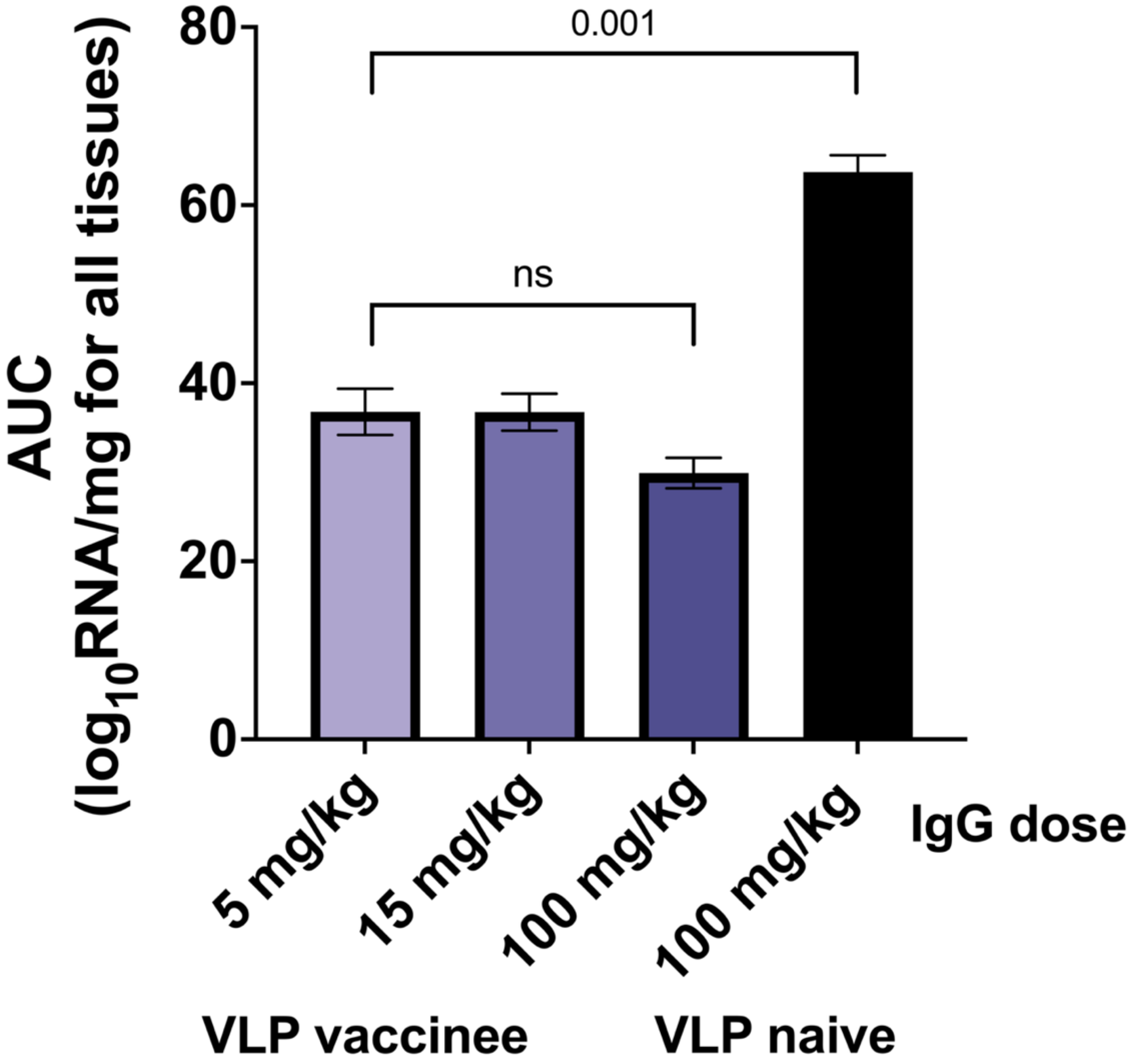

Supplemental Figure 22: Area under the curve (AUC) for CHIKV RNA levels in all sampled tissues after IgG administration from CHIKV VLP vaccinated or naïve people and CHIKV challenge in cynomolgus macaques. Analysis was performed on the combined log transformed viral RNA levels for all tissues and animals per study group. Bars represent mean + SEM. Tukey's multiple comparison test.

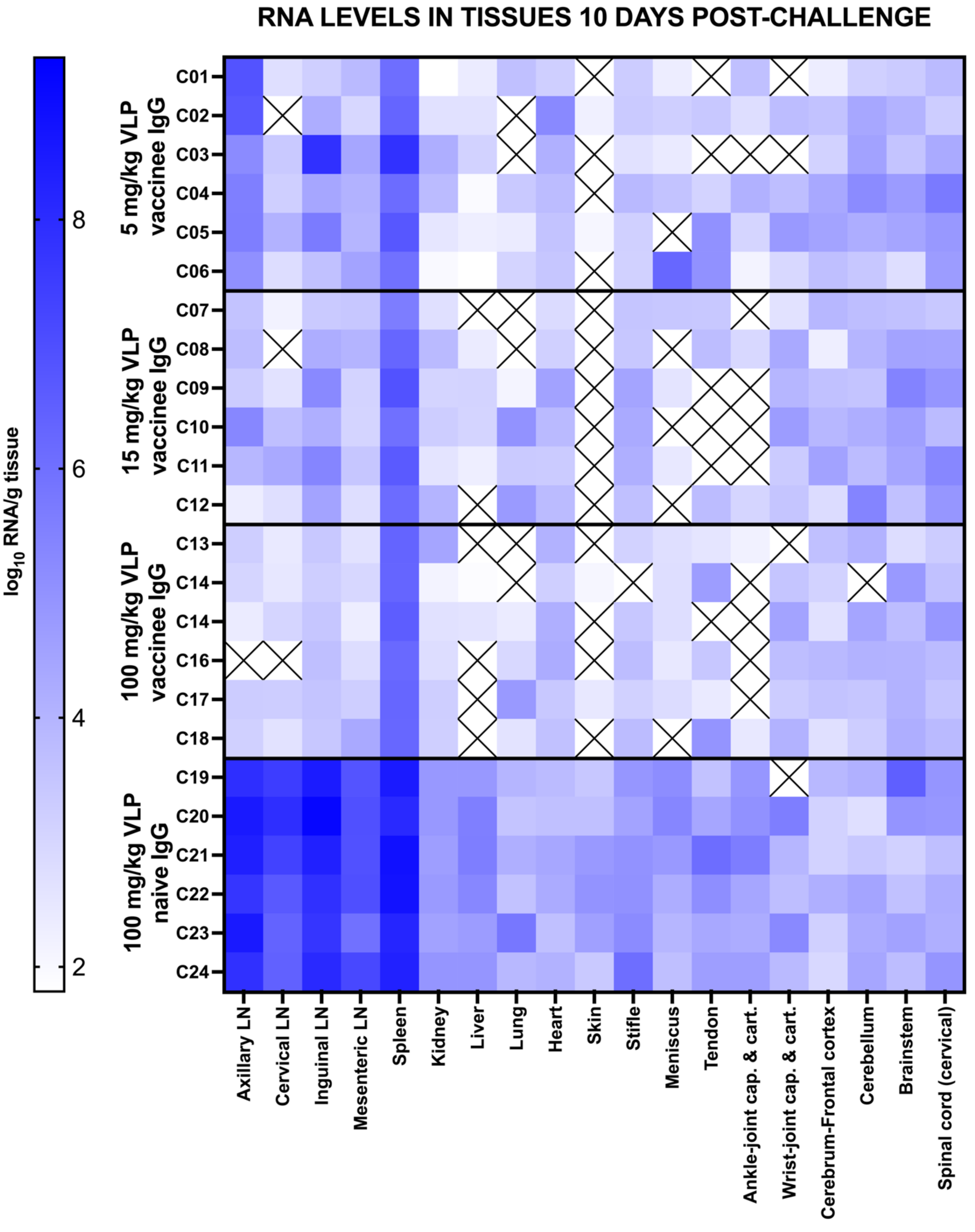

RNA LEVELS IN TISSUES 10 DAYS POST-CHALLENGE
log10 RNA/g tissue
2
4
6
8
5 mg/kg VLP vaccinee IgG
15 mg/kg VLP vaccinee IgG
100 mg/kg VLP vaccinee IgG
100 mg/kg VLP naive IgG
C01
C02
C03
C04
C05
C06
C07
C08
C09
C10
C11
C12
C13
C14
C14
C16
C17
C18
C19
C20
C21
C22
C23
C24
Axillary LN
Cervical LN
Inguinal LN
Mesenteric LN
Spleen
Kidney
Liver
Lung
Heart
Skin
Stifle
Meniscus
Tendon
Ankle-joint cap. & cart.
Wrist-joint cap. & cart.
Cerebrum-Frontal cortex
Cerebellum
Brainstem
Spinal cord (cervical)

Supplemental Figure 23: CHIKV RNA levels in all sampled tissues after IgG administration from CHIKV VLP vaccinated or naïve people and CHIKV challenge in cynomolgus macaques. CHIKV RNA levels were measured in homogenized tissues sampled at necropsy, 10 days post-challenge. An X symbol indicates tissue was not collected. LN is lymph node. Quads is quadricep. Cap. Is capsule. Cart. Is cartilage.

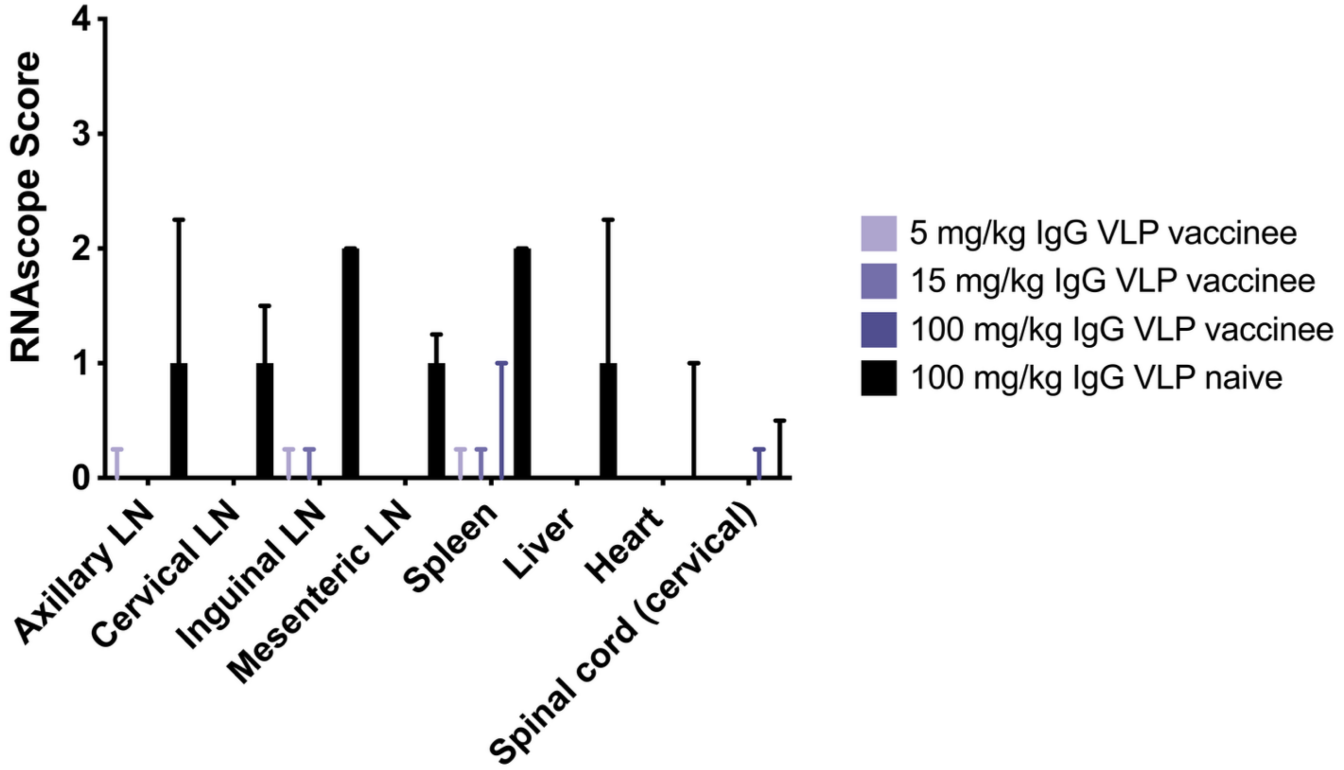

Supplemental Figure 24: CHIKV RNA in tissues after IgG administration from CHIKV VLP vaccinated or naïve people and CHIKV challenge in cynomolgus macaques detected using *in situ* hybridization (ISH). RNAscope ISH scoring criteria for lymphoid tissues are presented in Table 3. The graph shows mean values with error bars showing ranges. There were no differences between VLP vaccinee IgG dose groups ($p>0.05$). All scores for VLP vaccinee IgG treated groups were significantly lower than the control IgG group from VLP naïve persons ($p=0.002$) Kruskal-Wallis test.
